# Can Fascaplysins Be Considered Analogs of Indolo[2,3-*a*]pyrrolo[3,4-*c*]carbazoles? Comparison of Biosynthesis, Biological Activity and Therapeutic Potential

**DOI:** 10.3390/md24010018

**Published:** 2025-12-29

**Authors:** Maxim E. Zhidkov, Aleksandr M. Popov, Olga A. Soldatkina, Oleg A. Tryapkin, Lyubov N. Kharchenko

**Affiliations:** 1 Department of Chemistry and Materials, Institute of High Technologies and Advanced Materials, FEFU Campus, Far Eastern Federal University, Ajax Bay 10, Russky Island, 690922 Vladivostok, Russia; burnevskaia.oa@dvfu.ru (O.A.S.); triapkin_oa@dvfu.ru (O.A.T.); kharchenko.ln@dvfu.ru (L.N.K.); 2 Departments of Biotechnology and Marine Natural Compounds Chemistry, G.B. Elyakov Pacific Institute of Bioorganic Chemistry, Far Eastern Branch of The Russian Academy of Sciences, 690922 Vladivostok, Russia; popovam@piboc.dvo.ru

**Keywords:** staurosporines, rebeccamycins, fascaplysin, derivatives, biosynthesis, biological activity

## Abstract

For the first time, a comparative analysis has been conducted to elucidate the biosynthesis of three families of natural products—staurosporines/rebeccamycins, cladoniamides, and fascaplysins. Based on the available data, a well-founded hypothesis was formed that these metabolites arise through a shared biosynthetic pathway. A comparative evaluation of biological activity profiles and molecular mechanisms of action of the major representatives of these alkaloid families and their derivatives shows that, despite an apparent similarity between the activity spectra of indolo[2,3-*a*]pyrrolo[3,4-*c*]carbazoles and fascaplysins, they operate through different mechanisms. The biological effects of fascaplysin are driven primarily by the induction of metabolic stress rather than by the inhibition of DNA topoisomerase I or of a broad-spectrum protein kinases. The successful optimization of natural indolo[2,3-*a*]pyrrolo[3,4-*c*]carbazoles—compounds with initially poorer pharmacokinetic properties than those of fascaplysin—to drug-like candidates underscores the substantial pharmaceutical potential of the fascaplysin scaffold. Several existing fascaplysin derivatives, after the improvement of their pharmacokinetic characteristics, may serve as promising leads for the development of a new class of antibiotics.

## 1. Introduction

The study of the biological resources of the World Ocean is currently undergoing rapid expansion and providing an increasingly solid foundation for biotechnology and modern drug discovery strategies [1]. Natural bioactive compounds continue to represent an inexhaustible reservoir of therapeutics, owing to the remarkable diversity of their chemical structures and the breadth of their biological effects. Marine ecosystems are now viewed as particularly rich sources of new antitumor and antimicrobial agents capable of acting against cancer cell lines and antibiotic-resistant pathogenic microorganisms. The direction of further research and potential biomedical applications of a natural metabolite largely depend on its biological activity profile and the specific mechanism through which it acts at the molecular level. The pronounced biological activity exhibited by secondary metabolites from marine invertebrates often forms the basis for successful translation to clinical candidates. Chemical modification of these molecules to generate derivatives with therapeutic potential or as biochemical probes remains a vigorous and rapidly developing field at the interface of organic chemistry, biochemistry, and molecular biology [2,3,4,5,6].

**Figure 1:**
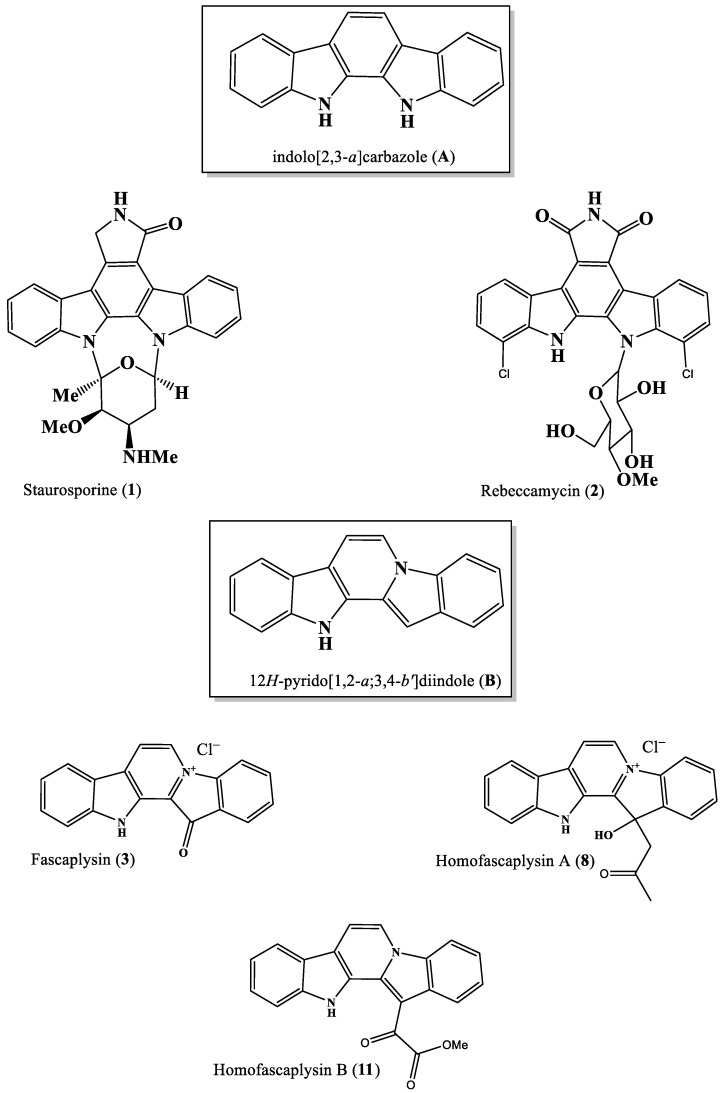
Structure of indolo[2,3-*a*]carbazole (**A**) and 12*H*-pyrido[1,2-*a*;3,4-*b*′]diindole (**B**), staurosporine (**1**), rebeccamycin (**2**), fascaplysin (**3**) and related alkaloids.

Within the vast structural diversity of known secondary metabolites, two groups of natural compounds sharing isomeric pentacyclic frameworks can be distinguished. The first group is based on the indolo[2,3-*a*]carbazole core (structure A), which includes tjipanazoles from terrestrial cyanobacteria as well as staurosporines and rebeccamycins obtained from terrestrial bacteria and various marine organisms. The second group features a 12*H*-pyrido[1,2-*a*;3,4-*b*′]diindole framework (structure B) and comprises the exclusively alkaloids of marine origin fascaplysins and homofascaplysins (Figure 1).

The number of known representatives of the first group is substantially greater, and their properties have been considerably more thoroughly investigated [7] (the diversity of natural indolo[2,3-*a*]carbazoles is also presented in Table S1 in the Supplementary Materials). Two major subclasses are recognized within the indolo[2,3-*a*]carbazoles–derivatives of the “key” indolocarbazoles staurosporine and rebeccamycin. There is also a smaller set of compounds lacking a pyrrolic ring. The earliest-discovered member of this family, staurosporine (STS, **1**), was isolated in 1977 from *Streptomyces staurosporeus*. Staurosporine analogs share two linkages between a carbohydrate fragment and indole rings and possess an amide group. About a hundred natural analogs have been reported from ascidians, mollusks, sponges, and various bacteria. Rebeccamycin (RB, **2**), isolated in 1983 from the actinomycete *Nocardia aerocolonigenes* (later *Lechevalieria aerocolonigenes*), became the defining representative of another large branch of indolo[2,3-*a*]carbazoles. Its distinguishing structural features include a single *N*-glycosidic bond and an imide group instead of an amide. To date, a lot of natural rebeccamycin analogs have been found in ascidians, sponges, and bacteria. Owing to their potent biological activities, many drug candidates derived from both staurosporine- and rebeccamycin-type indolo[2,3-*a*]carbazoles have been developed and studied as potential anticancer agents.

**Figure 2:**
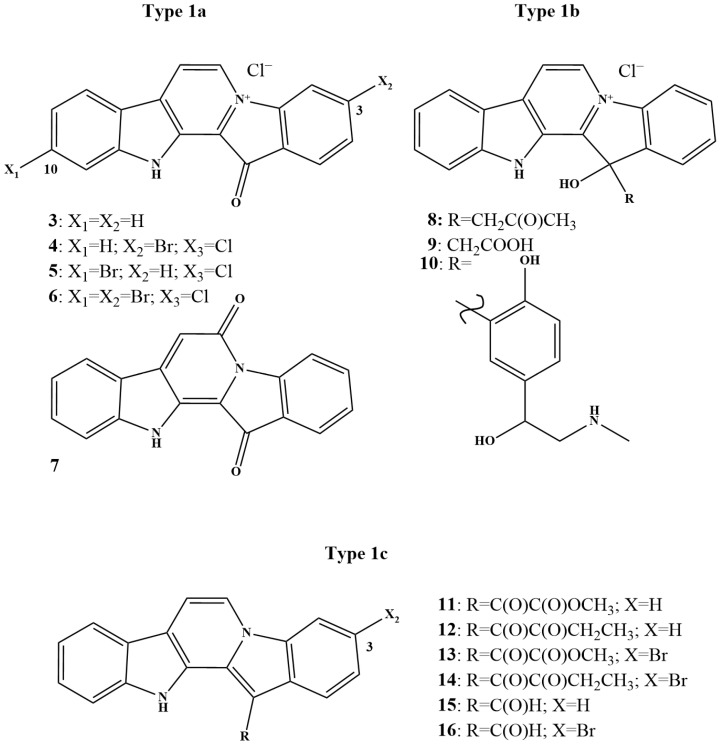
Structural diversity of fascaplysins.

In contrast, fascaplysin-type alkaloids are far less diverse and remain comparatively underexplored (Figure 2). Fascaplysin (Fas, **3**) and related compounds **4**–**16** are secondary metabolites isolated from tropical marine sponges of the genera Fascaplysinopsis, Smenospongia, and Thorectandra, as well as from ascidians and nudibranchs [8,9,10,11,12,13,14,15]. These metabolites include three subfamilies [9]. The first subtype comprises fascaplysin, its natural brominated derivatives (3-bromofascaplysin (**4**), 10-bromofascaplysin (**5**), 3,10-dibromofascaplysin (**6**)) and 6-oxofascaplysin (**7**). Homofascaplysins A include compounds formed formally by the addition of some fragments to the carbonyl group at C-13: homofascaplysin A (**8**), homofascaplysate A (**9**) and thorestandramine (**10**). The third subtype consists of homofascaplysins B and C (**11**–**16**). Their producers (sponges and ascidians) are consistently associated with characteristic inhabitants of coral reefs–hard corals of the genus Tubastraea. Sponges constitute the second most abundant group of sessile reef organisms after corals and zoantharians. Nudibranchs of the family Phyllidiidae feed Tubastraea-associated sponges, accumulate fascaplysin-type metabolites and employ them as chemical defenses in interspecific interactions within marine communities.

The present study includes the analysis of biosynthesis data for these classes of natural products. As a result, it was hypothesized that indolo[2,3-*a*]carbazoles are biosynthetic precursors of fascaplysins. A comparative analysis of the biological activity, mechanisms of action, and pharmacokinetic properties of them demonstrated that the therapeutic potential of derivatives of fascaplysin is comparable to, and in some respects exceeds, that of indolo[2,3-*a*]carbazoles.

## 2. Biosynthetic Relationship Between 12*H*-Pyrido[1,2-*a*;3,4-*b*′]diindoles and Indolo[2,3-*a*]carbazoles

**Scheme 1:**
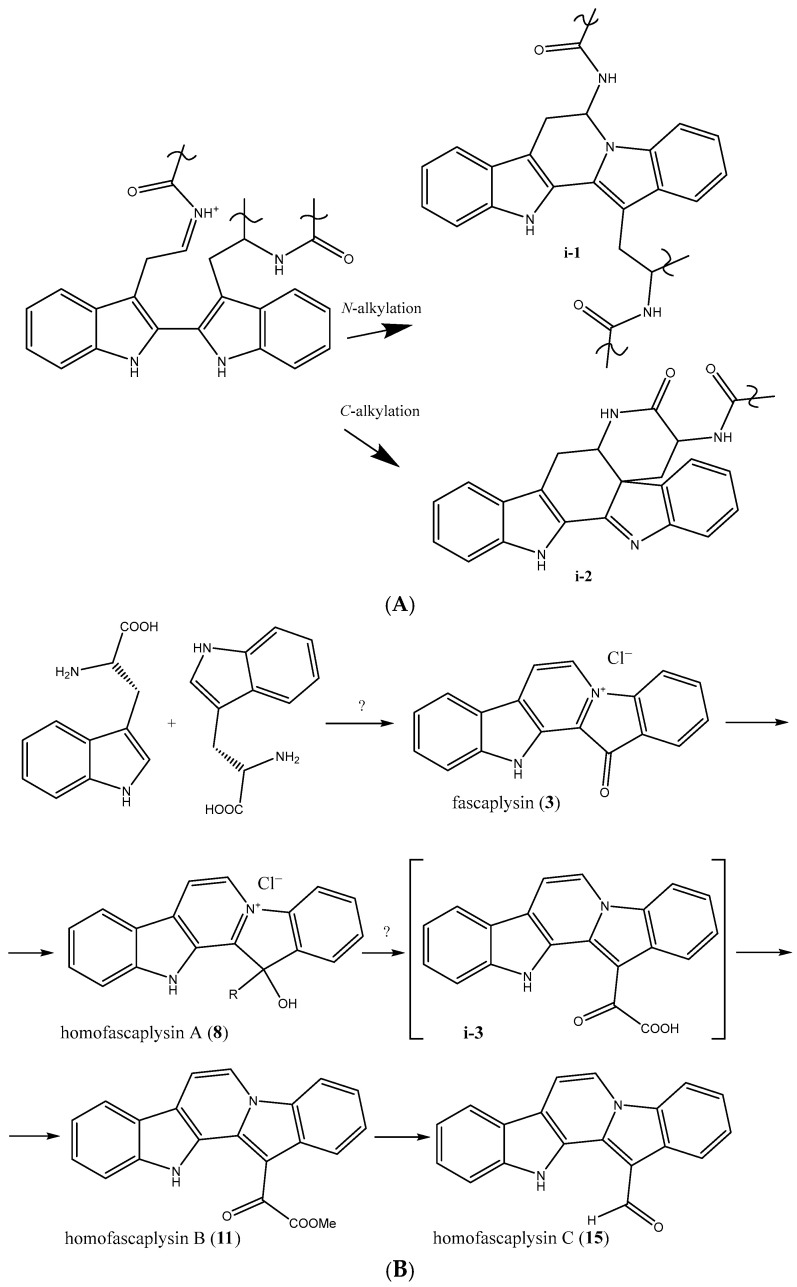
The first hypothesis about the biosynthetic relationship between natural 12*H*-pyrido[1,2-*a*;3,4-*b*′]diindoles and indolo[2,3-*a*]carbazoles (**A**) and putative biosynthetic pathways between tryptamines and fascaplysins (**B**).

Carter and Van Vranken previously proposed that peptides containing two tryptophan residues may first form intermediates through C2–C2′ coupling of their indole rings. According to their mechanistic model, these intermediates can follow two different pathways of cyclization. One pathway produces a 12*H*-pyrido[1,2-*a*;3,4-*b*′]diindole intermediate (**i**-**1**), and the other yields an indolo[2,3-*a*]carbazole intermediate (**i**-**2**) [16] (Scheme 1A). Later, the authors of [9] expanded this hypothesis and suggested a general biosynthetic pathway for fascaplysin alkaloids. In their model, bis-tryptophan precursors first give rise to fascaplysins **3**–**6**. After that, these compounds convert to homofascaplysins B (**11**–**14**) through homofascaplysin A intermediates. A subsequent decarboxylation produces homofascaplysins C (**15**–**16**) (Scheme 1B). The final step agrees with the known chemical reactivity of α-keto acids. However, the sequence of transformations fascaplysins → homofascaplysins A → homofascaplysins B raises doubts from the point of view of the mechanism of the second reaction.

A more plausible biosynthetic direction may have the opposite order. In this alternative sequence, homofascaplysin B converts to homofascaplysin C, and the latter then transforms into fascaplysin. The final step of this reverse pathway occurs readily under the action of peracids, as demonstrated by the synthesis of fascaplysin from homofascaplysin C (see Table S2 in the Supplementary Materials). However, this raises the question of the mechanism of conversion of the supposed intermediate **i**-**1** to homofascaplysin B. In this section, we will attempt to substantiate the possibility of the formation of homofascaplysin B from indolo[2,3-*a*]carbazoles through the intermediate formation of another class of natural compounds, cladoniamides, the biosynthesis of which is well known.

**Figure 3:**
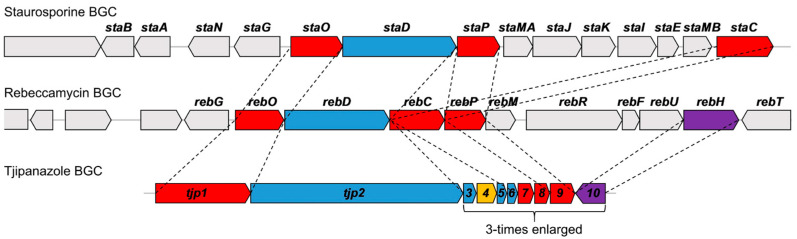
Comparison of the biosynthetic gene clusters of staurosporine, rebeccamycin and tjipanazole. Reproduced with modifications from [18].

Biosynthesis of staurosporines and rebeccamycins starts from tryptophan. Studies of their biosynthetic gene clusters confirmed this conclusion [17]. Later, the authors of [18] demonstrated extensive similarity between the biosynthetic gene clusters of staurosporine, rebeccamycin, and another class of natural compounds known as tjipanazoles (Figure 3).

**Scheme 2:**
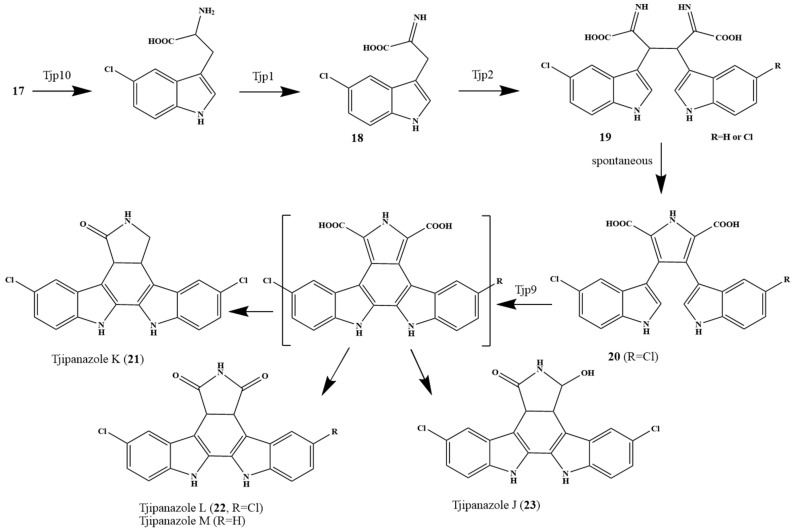
Scheme of the complete biosynthesis of tjipanazoles D and I from tryptophan. Reproduced with modifications from [18].

The biosynthesis of these metabolites begins with the oxidation of the amino group of tryptophan (**17**) to form imine **18** (Scheme 2). The oxidative coupling of two molecules of **18** produces intermediate **19**. The subsequent cyclization step forms the semiproduct–chromopyrrolic acid (**20**). Then specific enzymes catalyze the oxidative coupling of two indole rings. This reaction produces the pentacyclic indolo[2,3-*a*]carbazole core. At this stage, their biosynthetic pathways diverge to meet the structural requirements of different subclasses. The main modifications involve the pyrrolic ring and *N*-glycosylation. In [18], the biosynthesis of tjipanazole K (**21**), tjipanazole L (**22**), and tjipanazole J (**23**) is presented. Thus, the biosynthesis of three main structural skeletons of indolo[2,3-*a*]carbazoles (staurosporine, rebeccamycin, and UCN, respectively) is demonstrated.

**Figure 4:**
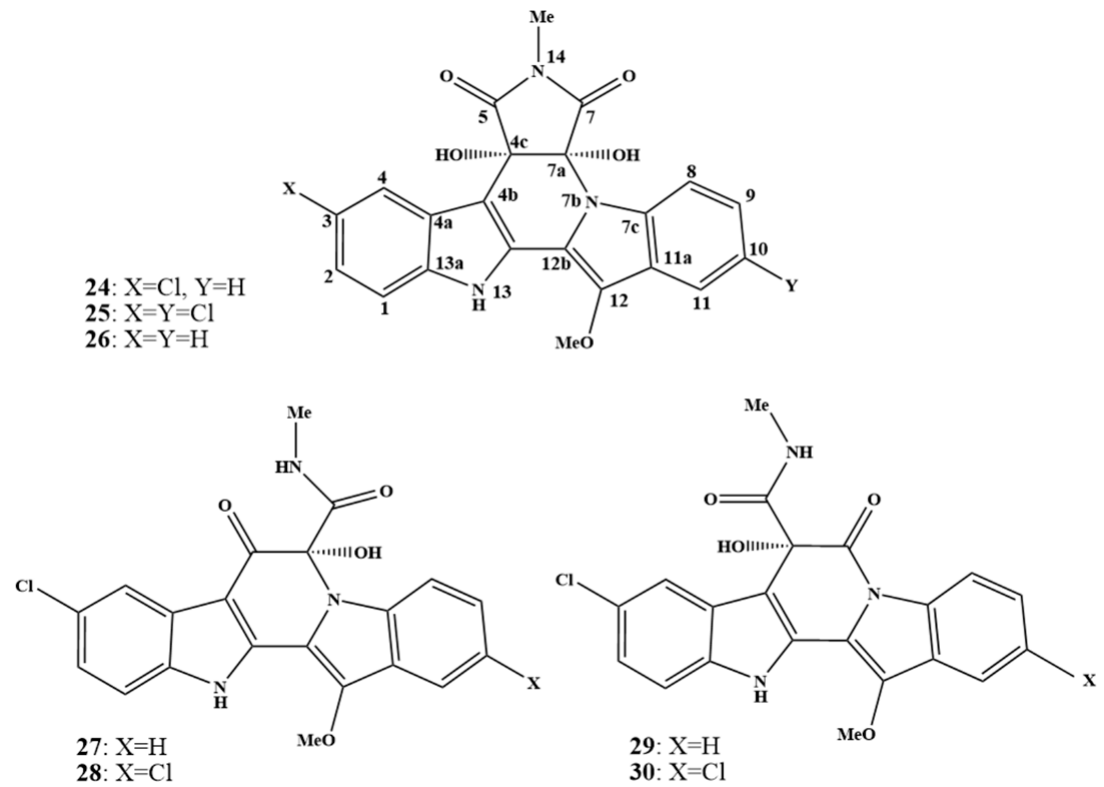
Diversity of cladoniamides A–G.

The question then arises: how does the conversation of indolo[2,3-*a*]carbazoles to 12*H*-pyrido[1,2-*a*;3,4-*b*′]diindoles? A small family of indolotryptaline alkaloids, cladoniamides A–G (**24**–**30**), isolated from *Streptomyces uncialis* (Figure 4), offers possible answers [19]. Some metabolites in this group act as potent inhibitors of HCT-116 colon cancer cell proliferation (IC_50_ = 0.08 μg/mL). This strong biological activity encouraged researchers to investigate their biosynthetic origin in more detail.

**Scheme 3:**
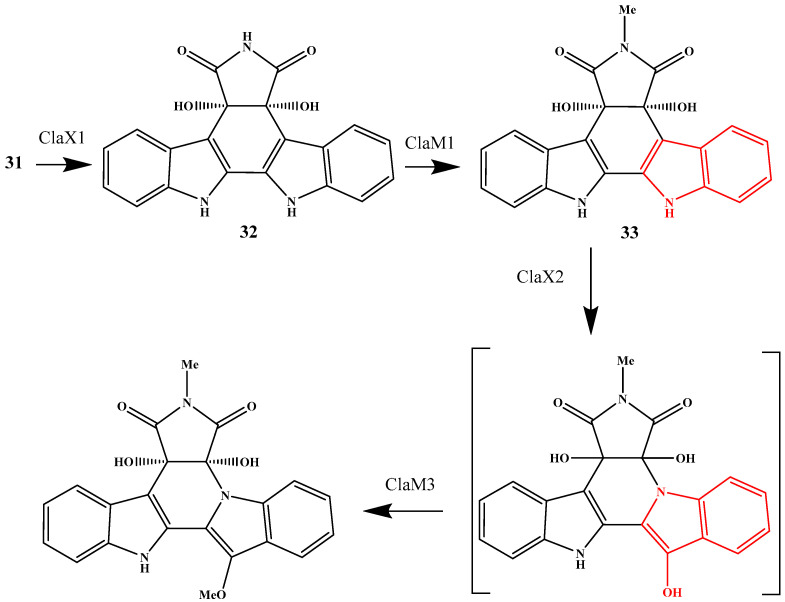
Biotransformation of indolo[2,3-*a*]carbazoles to indolotryptalines.

The authors of [20] identified the genetic cluster that converts indolocarbazoles such as arcyriaflavin A (**31**) into cladoniamides. The process begins with the formation of diol intermediate **32**. Its *N*-methylation and the rearrangement of the resulting intermediate **33** produce the target compounds (Scheme 3).

**Scheme 4:**
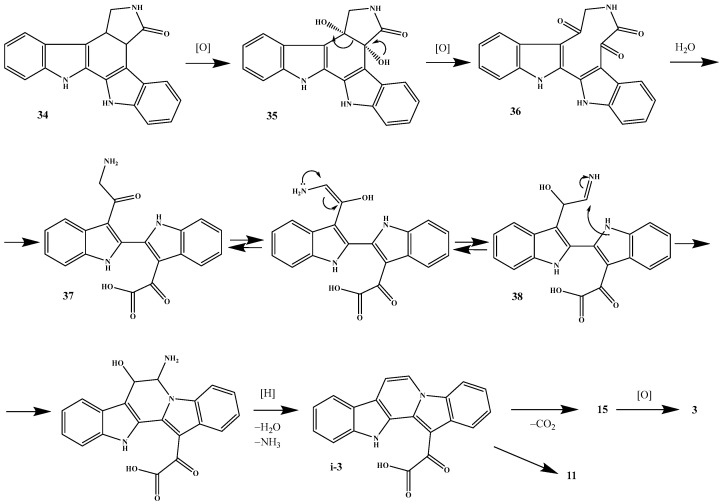
One of the possible mechanisms of biosynthesis of fascaplysin.

Based on the presented data, a putative mechanism for the biosynthesis of fascaplysin can be proposed (Scheme 4). The starting compound appears to be the staurosporine precursor K-252c (**34**). The key step involves oxidative cleavage of the C–C bond within the vicinal diol fragment of intermediate **35**. This cleavage forms two carbonyl groups and produces an unstable nine-membered macrocycle **36**. Hydrolysis of an amide bond opens the ring and generates intermediate **37**. The subsequent tautomerization forms imine **38**. Its cyclization occurs through the nitrogen atom of the second indole ring and results intermediate **i**-**3**, which represents the structural core of homofascaplysin B. Decarboxylation of this intermediate produces homofascaplysin C (**15**) that readily oxidizes to fascaplysin (**3**).

Since indolo[2,3-*a*]carbazoles are products of the bacterial genome, the proposed hypothesis indirectly indicates that the symbiotic bacteria are the original producers of fascaplysins. Further elucidation of the detailed mechanism of this biosynthetic sequence remains the important task for the specialists in the field of biochemical and genetic studies. Clarifying this mechanism may eventually allow to identify the natural producers of fascaplysin alkaloids.

## 3. Biological Activity of Fascaplysins and Indolo[2,3-*a*]carbazoles In Vitro

If we accept the biosynthetic relationship between natural indolo[2,3-*a*]carbazoles and pyrido[1,2-*a*;3,4-*b*′]diindoles, it is advisable to conduct a comparative analysis of their biological activity. Among indolo[2,3-*a*]carbazoles staurosporine was the first identified compound. STS displays the broadest range of biological effects. It exhibits cytotoxic, antifungal, and antiparasitic activities. In addition, STS demonstrates hypotensive, neuroprotective, and neurotrophic effects. It inhibits platelet aggregation, suppresses smooth muscle contraction [21], and blocks T-lymphoblast proliferation in response to mitogens [22]. Natural indolo[2,3-*a*]carbazoles produce pleiotropic effects, as summarized in previous reviews [7]. Most studies examine their cytostatic and cytotoxic effects against human and murine tumor cell lines. These studies include 26 natural derivatives and many synthetic analogs. These compounds also act as antibacterial agents, primarily against Gram-positive bacteria [23,24,25], and display antifungal activity [26,27]. Some derivatives demonstrate antiparasitic activity [28,29]. Antiviral effects have been reported against herpes simplex virus type II [30], human cytomegalovirus [31,32], and Epstein–Barr virus [33]. Additional biological effects include macrophage activation [34], inhibition of neuronal differentiation in response to nerve growth factor [35,36], and immunosuppression in vitro [37].

Among fascaplysin-type alkaloids, fascaplysin has been studied most extensively. Fas exhibits a broad spectrum of biological activities like those of indolo[2,3-*a*]carbazoles. It shows antibacterial [8,12,13], antifungal [8], antiviral (HIV-1 reverse transcriptase inhibition) [11,13], antiparasitic, and antimalarial activities [13]. Fas demonstrates potent cytotoxicity against diverse tumor cell lines at low concentrations [38]. Its cytotoxicity often occurs independently of tissue origin or chemoresistance profile. The inhibitory effect of Fas in vitro depends on cell type, compound concentration, and exposure duration. Early studies indicate that Fas strongly inhibits the proliferation of rapidly dividing tumor and microbial cells. It also exerts pronounced immunosuppressive effects. The combination of suppression of tumor cells and Gram-positive bacteria, together with inhibition of mitogen-induced lymphoid cell blastogenesis, suggests that Fas interferes with key cellular processes of proliferation and mitogenesis [39].

## 4. Mechanisms of Biological Activity of Indolo[2,3-*a*]carbazoles and Fascaplysins

Three main mechanisms of action have been identified for indolo[2,3-*a*]carbazoles: inhibition of a broad spectrum of protein kinases, inhibition of eukaryotic DNA topoisomerase I (Top1), and intercalation into DNA. The overall biological activity of each compound depends on the predominance of the specific mechanism and the cellular targets engaged. Minor structural modifications can shift the balance between mechanisms of action, strongly affecting biological profiles. Rebeccamycin derivatives primarily stabilize the Top1–DNA cleavage complex and intercalate into DNA [40,41]. However, anticancer activity does not always correlate directly with Top1 inhibition or DNA binding; additional targets, such as cyclin-dependent kinases, also contribute [41,42,43,44]. Some compounds in this class inhibit topoisomerase II [40,45]. Certain K252a derivatives act as nonselective protein kinase inhibitors while exhibiting Top2 inhibition [40].

**Figure 5:**
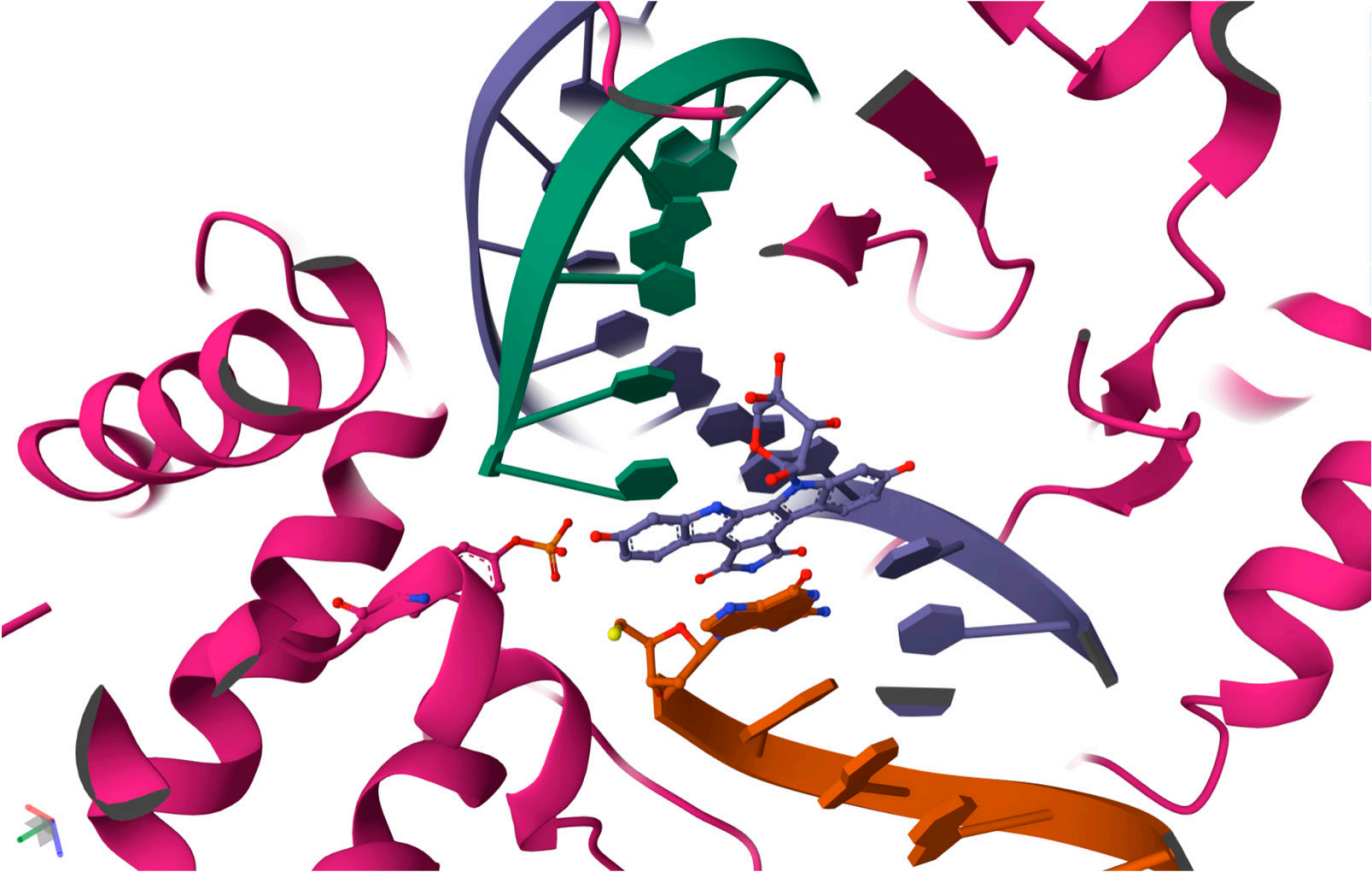
Structure of the crystalline complex of indolocarbazole SA315F with topoisomerase I and a DNA fragment (PDB ID 1SEU) [47]. Reproduced from RCSB PDB [48]. Mol* was used for image preparation [49].

The structure–activity relationship (SAR) studies allowed to link specific structural features of rebeccamycin derivatives with Top1 inhibition and DNA intercalation. As a result, three functional domains were identified: the imide group is important for topoisomerase binding, the planar chromophore provides DNA intercalation, and the carbohydrate fragment is necessary for minor- or major-groove insertion in DNA (Figure 5) [46,47]. Carbohydrates are critical for Top1 inhibition and DNA intercalation. Introducing the second linkage to the carbohydrate diminishes activity. Replacing a β-*N*-glycosidic bond with an α-*N*-glycosidic bond reduces both activities. Removing the carbohydrate entirely abolishes these functions. The presence of chlorine atoms on the planar aromatic core reduces DNA intercalation, whereas modifications at the imide nitrogen—polar (amino, formylamino, hydroxy) or nonpolar (methyl)—do not impede intercalation [41].

**Scheme 5:**
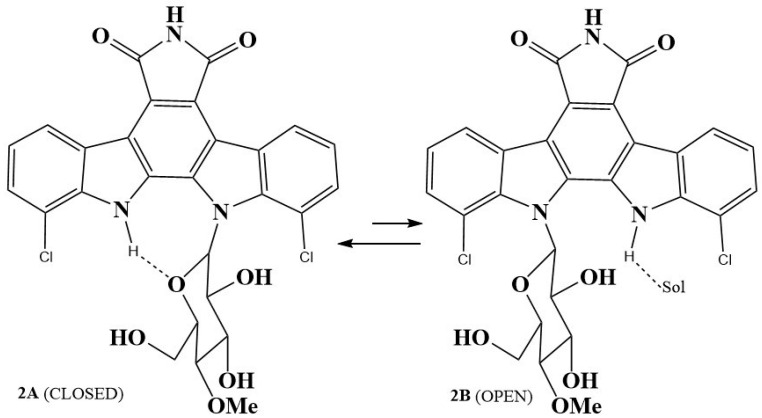
“Open” and “closed” conformations of rebeccamycin.

Conformational flexibility around the *N*-glycosidic bond establishes equilibrium between two conformations: closed 2A, with an intramolecular hydrogen bond between indole NH and pyranose oxygen, and open 2B, in which NH forms hydrogen bonds with solvent (Scheme 5) [50,51]. Methyl substitution destabilizes the closed conformation and alters the orientation of aglycone and sugar. Replacing the indole NH proton with an alkyl group reduces DNA binding and abolishes anti-Top1 activity, lowering cytotoxicity [51]. The lack of direct correlation between Top1 inhibition and cytotoxicity reflects the additional protein kinase activity of Top1, which phosphorylates proteins involved in spliceosome assembly and gene regulation [52]. Some antitumor indolo[2,3-*a*]carbazoles inhibit Top1 relaxation, bind DNA, and inhibit Top1 kinase activity [53,54].

**Figure 6:**
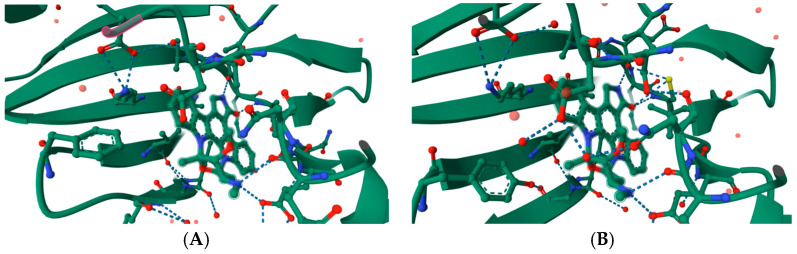
Structure of the crystalline complex of staurosporine with PDK1 (PDB ID 1OKY [56]) (**A**) and CDK1 (PDB ID 1NVR [70]) (**B**). Reproduced from RCSB PDB [48]. Mol* was used for image preparation [49].

Pharmaceutical interest in indolocarbazoles originated from staurosporine’s ability to inhibit protein kinase C (PKC) at nanomolar concentrations [55]. Subsequent studies revealed inhibition of multiple protein kinases, establishing STS as a potent, nonselective inhibitor [21]. Structural analyses of indolocarbazole–enzyme complexes showed competition with ATP at kinase ATP-binding pockets. The planar chromophore occupies the adenine-binding site, the carbohydrate engages the ribose-binding region through hydrogen bonding and hydrophobic interactions, and the amide group hydrogen-bonds with the N- and C-terminal linker (Figure 6). Hydroxyl and amino groups at C-6 and C-7 enhance binding through additional hydrogen bonds [56]. Consequently, staurosporine-type molecules effectively mimic ATP geometry and hydrogen-bonding and enable inhibition of broad spectrum of kinases [57]. Their targets include ABC transporters such as P-glycoprotein and ABCG2, involved in multidrug resistance [58,59]. The multitarget nature explains divergent biological effects: one molecule may induce cancer cell death, while a closely related analog may be neuroprotective. Selective inhibitors have also been developed through structural modification of the indolo[2,3-*a*]carbazole scaffold and include inhibitors of trkA tyrosine kinase [60], PKC isoforms [61,62], CDKs [63,64,65,66], GSK-3β [67], VEGFR2 [68], MLKs [69], and CMV pUL97 kinase [31].

**Figure 7:**
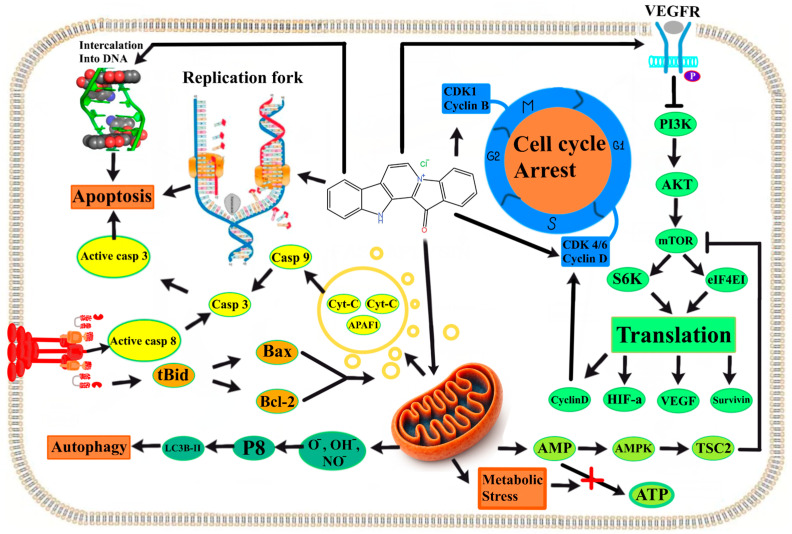
The main mechanisms of action of fascaplysin. Shown are intercalation into DNA, effects on the cell cycle (dark blue), the PI3K–AKT–mTOR signaling cascade (different shades of green), the development of apoptosis via the extrinsic and mitochondrial pathways (yellow), and of autophagy (magenta).

Between 2000 and 2010, four main mechanisms of action of fascaplysin were identified. It initially attracted attention due to selective inhibition of CDK4, a G1→S cell-cycle regulator, with IC_50_ = 0.35 μM in vitro [71]. Studies of Fas binding to calf thymus DNA revealed a two-site model, K_1_ = 2.5 × 10^6^ M^−1^, K_2_ = 7.5 × 10^4^ M^−1^, indicating DNA intercalation contributes to its spectrum of action [72]. Intercalation usually suggests potential nonspecific cytotoxicity that motivated to design of nonplanar Fas analogs without the planar pentacyclic pyridodiindole core but retaining CDK4 inhibition [73,74,75,76,77,78]. Fas also exhibits antiangiogenic effects. It inhibits human umbilical vein endothelial cells (HUVEC’s) proliferation more effectively than tumor cells BEL-7402 and reduces VEGF expression [79]. Cellular responses include G1 arrest in this case, confirmed by decreased CDK4, cyclin D1, and pRb Ser795 phosphorylation, and apoptosis via both extrinsic and mitochondrial pathways [80,81]. The ability to activate metabolic stress sharply distinguishes fascaplysin from indolo[2,3-*a*]carbazoles, for which this type of activity is not typical. Further studies refined and expanded the principal mechanisms of action of this alkaloid (Figure 7).

Computational studies showed the positive charge of Fas determines target selectivity for CDK4 [82]. However, in a comparative assessment of 178 low-molecular inhibitors against 300 major human kinases fascaplysin demonstrated focused, but weak inhibitory effects. For example, residual activity of the CDK4/D1 complex in the presence of 0.5 μM fascaplysin was 63.40%, whereas it was 5.05% for staurosporine. The largest inhibitory effect of fascaplysin was observed toward the CDK1/B complex (53.47%) [57]. In experiments on wild-type and Rb-knockout lung cancer cells fascaplysin showed equally high efficacy and exhibited stronger cytotoxicity than selective CDK4 inhibitors such as PD0332991 and LY2835219 [83]. This proves that CDK4 inhibition is not the primary determinant of antitumor action of fascaplysin.

The contribution of DNA-intercalation was evaluated using bulky *tert*-butyl Fas derivatives at C-6 and C-7, which showed reduced intercalation but retained cytotoxicity against drug-resistant prostate cancer cells [84]. Experiments also revealed pronounced slowing of replication fork progression. Overall, these data indicate that these compounds induce replication stress, which ultimately converts to toxic double-strand DNA breaks and causes cell death via caspase-independent apoptosis. It is possible that fascaplysin exerts similar effects, but this requires further study.

Induction of mitochondria-dependent apoptosis by fascaplysin was confirmed by showing the compound’s ability to generate reactive oxygen species (ROS) in small cell lung cancer (SCLC) NCI-H417 cells [85]. Addition of the antioxidant *N*-acetyl-*L*-cysteine to the medium to quench ROS resulted in a twofold decrease in Fas cytotoxicity. This indicates that metabolic stress is a major contributor of fascaplysin cytotoxicity. In HL-60 cells, in addition to the effects noted above, Fas caused loss of mitochondrial transmembrane potential, suppressed the PI3K–AKT–mTOR signaling cascade and induced autophagy [86].

It is well known that the PI3K–AKT–mTOR cascade is the primary regulator of cellular translation and integrates numerous biochemical pathways [87]. Its inhibition may be a direct consequence of oxidative stress, causing AMP accumulation, sequential activation of AMPK and TSC2, and, consequently, mTOR blockade. In [88], it was shown that fascaplysin induces AMPK activation. Alternative mechanisms are also possible, for example, Fas inhibits VEGFR3, VEGFR2 and tropomyosin receptor kinase A (TRKA) with IC_50_ values of 2–3 μM by a non-ATP-competitive and DFG-independent mechanism [83]. Through blockade of the mTOR–4E-BP1–p70S6K1 pathway, Fas suppresses *cap*-dependent translation of a range of proteins essential for cell function, including the antiapoptotic factor survivin, hypoxia-inducible factor-1α (HIF-1α), transcriptional regulator c-Myc, cell cycle regulator cyclin D1 and VEGF. This explains many of the observed effects of fascaplysin, including cell cycle arrest and antiangiogenic properties.

The mTOR pathway is also often responsible for autophagy induction. However, in the case of fascaplysin, p8 (nuclear protein 1, NUPR1) is a key regulator in VEC autophagy, and it mediated VEC autophagy via mTOR independent pathways [89]. This protein promotes the accumulation of ROS and is included in the autoregulatory loop with p53, which, when located in the nucleus, is an inducer of autophagy. The mechanism of Fas-induced autophagy appears to be protective, because in the presence of the autophagy inhibitor 3-methyladenine Fas more strongly inhibited proliferation and induced apoptosis compared with treatment with Fas alone.

Among other mechanisms of action of fascaplysin it is necessary to note the ability of fascaplysins **3** and **5** to modulate μ-opioid receptors with EC_50_ values of 6.3 and 4.2 μM, respectively, acting as balanced agonists and thus resembling endogenous opioid peptides such as endorphins [90]. Additionally, Fas noncompetitively inhibits acetylcholinesterase (with ∼60-fold greater affinity for AChE than for butyrylcholinesterase) [91] and activates P-glycoprotein, which has driven the separate area of research of fascaplysin derivatives [92].

## 5. Comparation of Properties of Natural Indolo[2,3-*a*]carbazoles and Fascaplysin In Vivo

**Figure 8:**
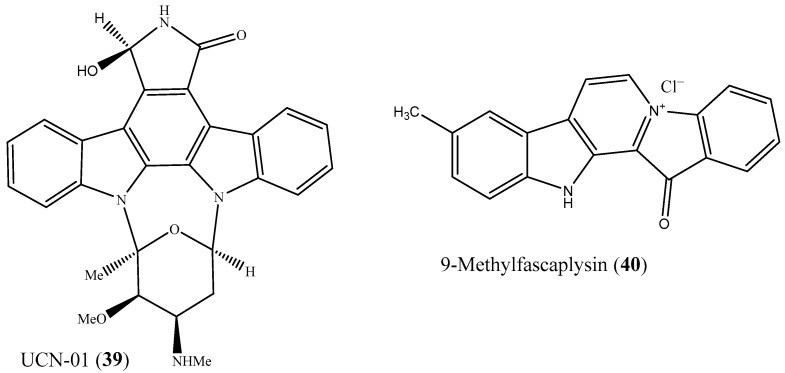
Structures of UCN-01 (**39**) and 9-methylfascaplysin (**40**).

Numerous studies have evaluated the antitumor activity of natural indolo[2,3-*a*]carbazoles toward transplantable tumor and xenograft models [23,34,45,93,94]. For example, the staurosporine analog UCN-01 (**39**, Figure 8) increased mean survival time (MST) in a mouse P388 leukemia model by 24% at a single intravenous dose of 15 mg/kg, whereas staurosporine itself was ineffective under these conditions [94]. Rebeccamycin was tested in P388 and L1210 leukemia models and B16 melanoma; doses of 8–16 mg/kg increased survival by 125–136%, while escalation to 256 mg/kg provided only modest additional gains (136–175%) [93]. We were the first to evaluate the antitumor efficacy of Fas in vivo and found that doses from 5 to 20 mg/kg did not significantly increase MST against ascitic Ehrlich carcinoma model [39]. Subsequent solid tumor models demonstrated improved efficacy: A375 tumors responded to 1 mg/kg with a threefold tumor volume reduction [83]; HCT 116 tumors exhibited 42% tumor growth inhibition (TGI) at 4 mg/kg [95]; S180 tumors showed 32.6% TGI at 5 mg/kg [96].

For application in vivo, toxicity to blood components is important. Exposure of erythrocytes to 5 μM Fas for 48 h induced eryptosis without significant hemolysis [97]. At concentrations up to 10 μM, Fas did not affect platelet survival, while 25 μM reduced survival to 80%. Fas decreased ERK phosphorylation, did not alter P-selectin expression, but markedly inhibited integrin GPIIb/IIIa activation—an antithrombotic target—reducing platelet aggregation at 10 μM in vitro. In vivo, Fas prolonged vessel occlusion and tail bleeding times by 40–50% relative to controls, though less than heparin [98].

Pharmacokinetic profiles are particularly relevant. UPLC–MS/MS methods detect Fas in rat plasma following intravenous administration with a 1 ng/mL detection limit, which made it possible to study the pharmacokinetics of Fas and its 9-methyl derivative (**40**, Figure 8) [99,100]. Table 1 summarizes the pharmacokinetic parameters of compounds **3** and **40**, compared with another known cytotoxic antibiotic, doxorubicin (Dox) [101]. Plasma C_max_ values of Fas and 9-methylfascaplysin after intravenous administration are several-fold lower than those of Dox, likely due to stronger plasma protein binding. Compound **40** binds less strongly than the parent molecule. Oral administration reduces differences in C_max_, reflecting higher oral bioavailability of Fas (27.2%) and **40** (18.3%) versus Dox (8.6%). Total clearance, elimination rate constant, and half-life indicate that 9-methylfascaplysin is eliminated more slowly than Fas, at rates comparable to Dox, whereas Fas undergoes more rapid clearance. Distribution volume (V_d_) values show that Fas and derivative **40** distribute more extensively into peripheral tissues relative to Dox.

Overall, these data indicate that Fas and 9-methylfascaplysin exhibit pharmacokinetics generally like Dox, though not optimal. Nonoptimized natural indolo[2,3-*a*]carbazoles display inferior pharmacokinetics; for instance, rebeccamycin has very poor solubility, and staurosporine was sequestered 97.5–98.8% in lungs and heart during first pass in rats [102]. This indicates the greater initial potential of fascaplysin for pharmacokinetic optimization compared to indolo[2,3-*a*]carbazoles.

## 6. Lead Candidates Based on Indolo[2,3-*a*]carbazoles and 12*H*-Pyrido[1,2-*a*;3,4-*b*′]diindoles

Over the past several decades, numerous indolo[2,3-*a*]carbazole derivatives with antitumor properties have been synthesized and evaluated. Among staurosporine-derived compounds, UCN-01 (7-hydroxy-staurosporine; KW-2401, NSC-638850, **39**, Figure 8) is notable for inhibiting multiple protein kinases, including PKC isoforms, cyclin-dependent kinases, and PDK1, thereby exerting broad antineoplastic activity. UCN-01 also enhances the cytotoxic effects of DNA-damaging agents and antimetabolites. It was clinically tested for leukemias/lymphomas, advanced solid tumors, melanoma, and small-cell lung cancer [103]. However, its human pharmacokinetics differed significantly from preclinical models due to extremely high-affinity binding to α1-acid glycoprotein (AAG), that limited free plasma concentrations and impeded its further clinical development.

**Figure 9:**
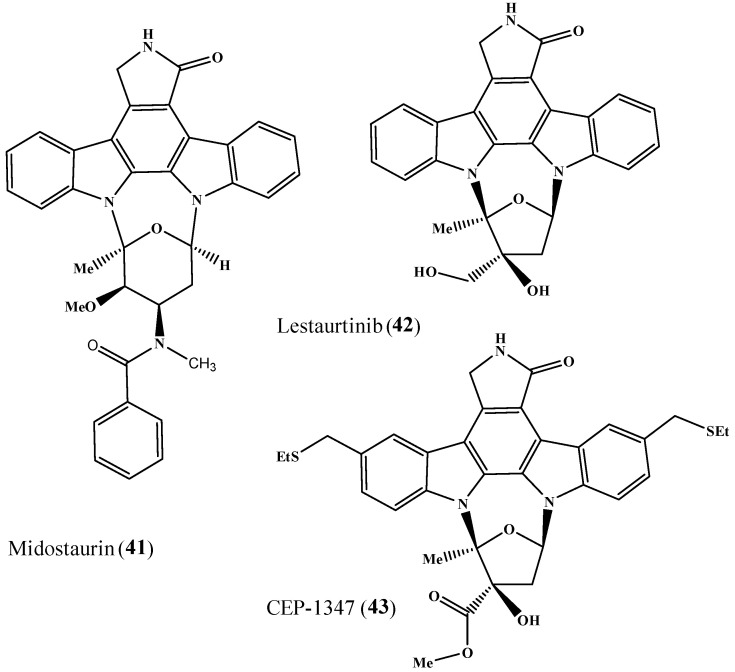
Structures of midostaurin (**41**), lestaurtinib (**42**) and CEP-1347 (**43**).

Midostaurin (PKC412, CGP-41251, 4-*N*-benzoyl-STA, **41**, Figure 9), an *N*-benzoyl staurosporine derivative, is a potent inhibitor of activated FLT3 kinase and other kinases, including PDGFR-α/β, CDKs, Src, Fgr, Syk, KIT and VEGFR [104]. Activating mutations in FLT3, particularly internal tandem duplications (FLT3-ITD), confer a poor prognosis in acute myeloid leukemia (AML). Clinical trials led to FDA and EMA approval of midostaurin for AML with activating FLT3 mutations. Midostaurin is also approved for advanced systemic mastocytosis due to its inhibitory activity against mutant KIT forms [105,106].

Lestaurtinib (**42**) inhibits FLT3 by suppressing its autophosphorylation and also demonstrates JAK2 inhibition. Lestaurtinib displayed cytotoxicity in vitro and antileukemic activity in vivo for models bearing activating FLT3 mutations [107]. It also suppresses proliferation and JAK2/STAT5 signaling in CD34+ erythroid cells from patients with myeloproliferative disorders, without affecting erythropoiesis in healthy controls [108]. However, clinical trials showed that lestaurtinib monotherapy had limited efficacy in AML, regardless of FLT3 mutation status [109,110,111]. Addition of lestaurtinib to induction chemotherapy did not improve 5-year overall or relapse-free survival [111]. Trials of lestaurtinib in myelofibrosis were terminated in phase II due to limited efficacy and frequent adverse events [112]. CEP-1347 (KT-7515, **43**), another derivative of metabolite K252a, displays neuroprotective properties and potent MLK inhibition [69]. CEP-1347 entered phase II/III clinical trials to evaluate delay of disability progression in Parkinson’s disease [113], but the trial was terminated after interim analysis showed a lack of benefit [114].

**Figure 10:**
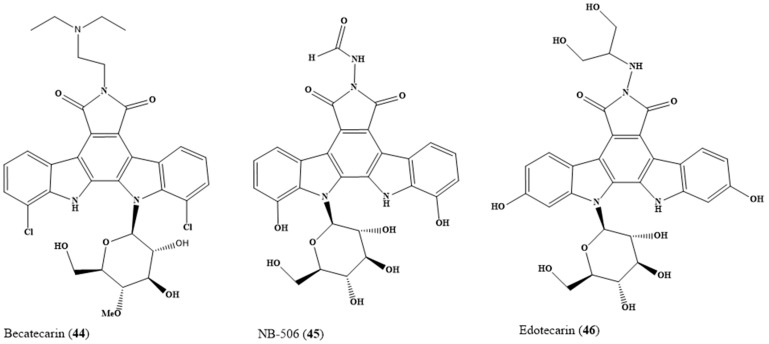
Structures of the rebeccamycin derivatives becatecarin (**44**), NB-506 (**45**) and edotecarin (**46**).

As rebeccamycin is practically insoluble in water, its optimization was focused on obtaining more soluble analogs. Becatecarin (6-*N*-(diethylaminoethyl)-rebeccamycin, **44**, Figure 10) intercalates into DNA and inhibits topoisomerases I and II. In preclinical studies it showed antitumor activity in P388 leukemia and B16 melanoma models [115]. Phase II studies have shown moderate efficacy of becatecarin for metastatic renal cancer [116], refractory breast cancer [117], neuroblastoma and rhabdomyosarcoma in children [118]. Becatecarin proved insufficiently effective in phase II trials for small cell and non-small cell lung cancers [119,120].

The 6-*N*-formylamino derivative of metabolite BE-13793C NB-506 (**45**) was more water-soluble, inhibited Top1 relaxation, bound DNA, and inhibited Top1 kinase activity, suppressing growth of diverse tumor lines at ≤2 μM with selective cytotoxicity [121]. NB-506 showed antitumor activity in M5076 and Ehrlich solid tumors and caused regression of PC-13 lung nodules, MKN-45 gastric tumor and HCT-116/LS-180 colon tumors [122]. NB-506 had low cumulative toxicity, supporting clinical investigation initiated in 1994, but subsequent development shifted to edotecarin (**46**), differing by repositioning two hydroxyls on the chromophore and replacing the formylamino substituent with a 1,3-dihydroxypropyl group [123]. Edotecarin more effectively stabilizes the Top1–DNA complex, generating single-strand breaks, and outperforms camptothecin at the molecular level. Despite promising preclinical data and synergistic combinations with cisplatin, 5-FU, etoposide, paclitaxel, doxorubicin, vincristine, CPT and gemcitabine in vitro, edotecarin failed to demonstrate sufficient clinical efficacy and its trials were discontinued [124].

**Scheme 6:**
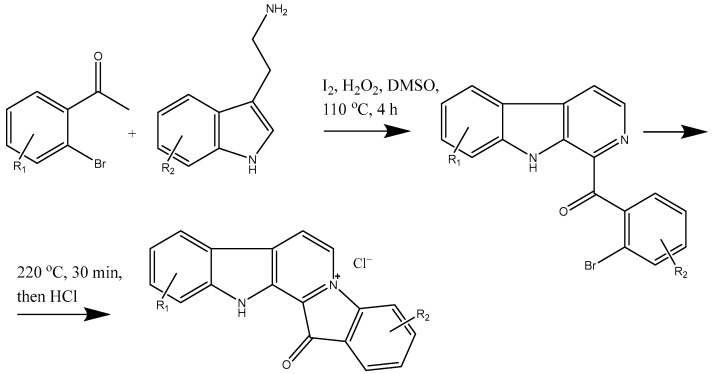
The main scheme for the synthesis of fascaplysin and its derivatives.

Studies of derivatives of fascaplysin have been limited by their low availability. Since the first isolation numerous approaches of the synthesis of fascaplysin have been developed [91,125,126,127,128,129,130,131,132,133,134,135], but the variety of known fascaplysin derivatives is limited mainly mono- and dihalogenated analogs at rings A and E and simple monoalkyl homologues at rings A and C [38]. Among various methodologies, the most commonly employed is a two-stage approach which includes the multicomponent condensation of suitably substituted tryptamines and acetophenones in the presence of molecular iodine in DMSO and high-temperature quaternization of resulting 1-benzoyl-β-carboline to target fascaplysin [132] (Scheme 6, other approaches are presented in Table S2 in the Supplementary Materials).

**Figure 11:**
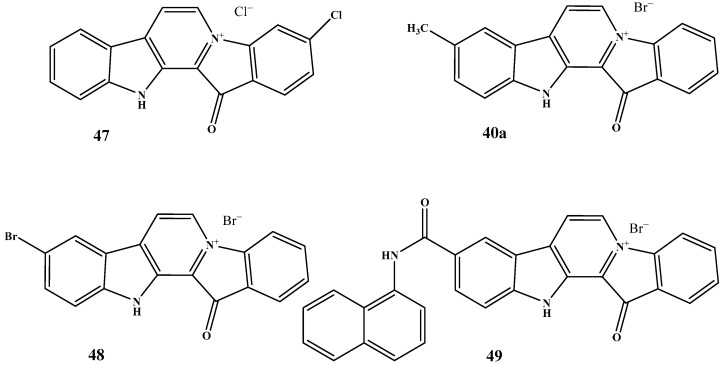
Structure of the most promising monoderivatives of fascaplysin.

Among mono-substituted Fas analogs, 3-chlorofascaplysin (**47**, Figure 11) induces apoptosis via metabolic stress and exhibits antiangiogenic activity, reducing Ehrlich carcinoma tumor growth by 30% at 7 mg/kg over 7 days [136]. 9-Methylfascaplysin (bromide as a counter anion, **40a**) inhibits acetylcholinesterase, suppresses β-amyloid fibril formation and protects neuronal cells from oligomeric Aβ- and H_2_O_2_-induced toxicity in vitro. In hippocampal models, both Fas and 9-methylfascaplysin attenuated scopolamine-induced cholinergic impairment and prevented Aβ oligomer-induced cognitive deficits in vivo [137,138]. 9-Methylfascaplysin (**40a**), 9-bromofascaplysin (**48**), and amide derivative **49** displayed potent activity against MRSA ATCC43300, with inhibition exceeding that of vancomycin by an order of magnitude [139]. Mechanistically, these compounds disrupted cell wall integrity and membranes. Compound **49** at 1.56 μg/mL inhibited 78.9% of MRSA biofilm formation over 24 h, exhibited low hemolysis, and limited mammalian cytotoxicity. In a murine peritonitis model, compound **49** resulted in 20% survival over 7 days at 10 mg/kg.

**Figure 12:**
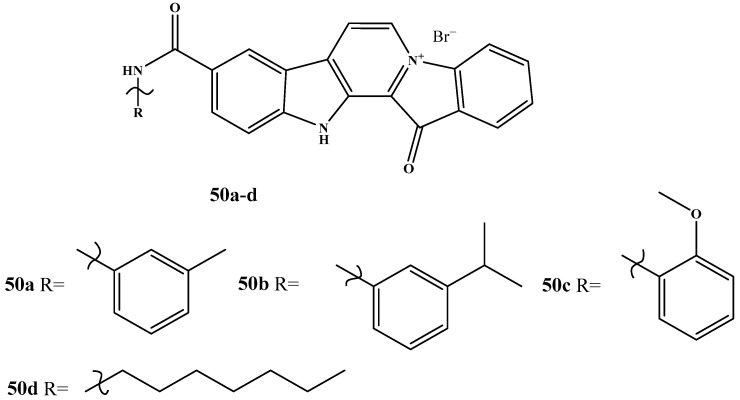
Structures of fascaplysin derivatives **50a**–**d**.

The optimization of structure of amide **49** led to production of a series of its analogs **50a**–**d** (Figure 12). Among them compound **50a** promoted polymerization of the filamentous temperature-sensitive mutant Z (FtsZ) at 4 μg/mL and compound **50c** produced a 40% reduction in FtsZ GTPase activity at 0.32 μg/mL. Thus, antibacterial activity of these derivatives is at least partly due to interaction with FtsZ [140]. FtsZ, a prokaryotic homolog of eukaryotic tubulin encoded by *ftsZ*, assembles into the Z-ring at the future division site and is an attractive target for addressing the global rise in antibiotic resistance [141,142,143,144].

**Figure 13:**
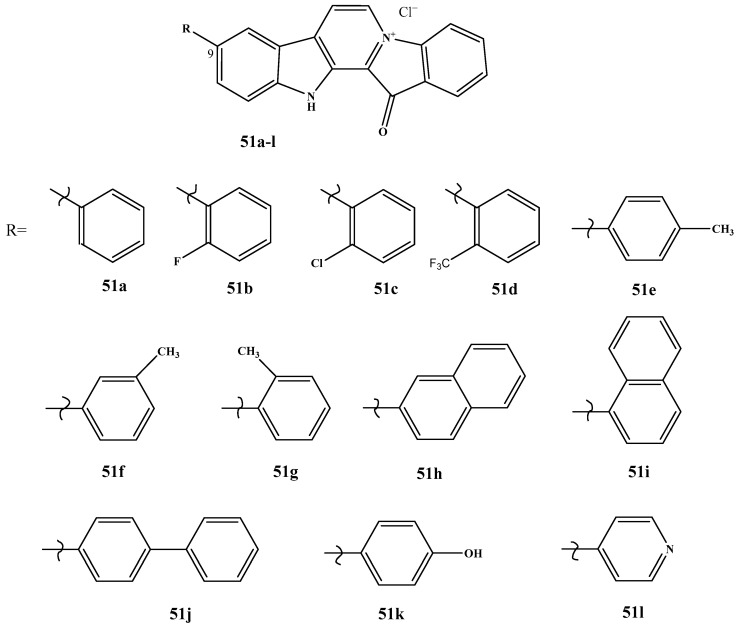
Structure of 9-aryl-substituted fascaplysin derivatives **51а**–**l**.

Further aryl substitution at C-9 generated derivatives **51a**–**l**, exhibiting potent bactericidal activity against Gram-positive (MIC = 0.024–6.25 μg/mL) and Gram-negative (MIC = 1.56–12.5 μg/mL) bacteria (Figure 13). The dual mechanisms of their action also included membrane disruption and inhibition of FtsZ polymerization [145]. The positional placement of phenyl substituent at C-9 was critical; adjacent positioning reduced activity [146]. In vivo, compound **51a** showed ED_50_ = 0.48 mg/kg in acute sepsis, ~9-fold lower than vancomycin and comparable in activity to fascaplysin. This indicates that a key limitation is rapid inactivation or removal from the circulation or tissues. Introduction of polar fragments to the phenyl substituent to increase solubility led to sharp loss of target activity in vitro; increasing volume of lipophilic substituent also failed to improve potency [146].

The comparative evaluation of 3-bromofascaplysin (**4**), 10-bromofascaplysin (**5**), 3,10-dibromofascaplysin (**6**), and a series of their isomers and analogs revealed that substitution at C-3 enhanced antimicrobial potency, while dual bromination at C-2 and C-9 further enhanced the antimicrobial properties by 4 to 16 times, depending on the tested strain. 3,10-Dibromofascaplysin inhibited 49.2% of tumor growth against subcutaneous Ehrlich carcinoma after 20 days of the experiment [147]. These data alongside its wide therapeutic window [148], selective cytotoxicity for prostate tumor cells (SI 7–9) [149], nanomolar leukemia cell inhibition via S/G2 arrest [150], and synergy with olaparib, platinum-based drugs, and taxanes [151], allow to consider 3,10-dibromofascaplysin, as well as 9-phenylfascaplysin (**51a**) as lead candidates for further therapeutic development.

## 7. Conclusions

The accumulated evidence indicates that marine alkaloids related to fascaplysin, as well as rebeccamycins, staurosporines, and cladoniamides, likely originate from a single bacterial biosynthetic cluster evolved to produce highly bioactive metabolites. Experimental confirmation of this hypothesis via genetic approaches remains necessary. These alkaloids are bisindole derivatives, which are known to often outperform their monomeric precursors, which also indicates the significant importance of studying such compounds [152]. Fas, STS, RM, and their derivatives exert potent and often nonselective effects on essential biochemical processes, providing a competitive advantage to the producing organisms in natural ecosystems and play roles in the natural functioning of symbiotic communities. Consequently, these indole derivatives have potential not only as therapeutic agents with antitumor, antimicrobial, immunomodulatory, and other activities but also as tools to modulate interspecies and interkingdom interactions in terrestrial and marine ecosystems, including within the human microbiome [153].

Many drug candidates derived from indolo[2,3-*a*]pyrrolo[3,4-*c*]carbazoles have undergone clinical evaluation. A major advantage of indolocarbazoles is their ability to target multiple intracellular pathways, enabling simultaneous activation of various tumor cell death mechanisms and potentially limiting the emergence of drug resistance. However, this pleiotropy increases the risk of adverse effects, reduces the therapeutic index, and may compromise advantages relative to existing therapies. Therefore, balancing efficacy and safety is critical for successful drug development. The development of antitumor agents based on protein kinase inhibitors and Top1/DNA intercalators derived from indolo[2,3-*a*]carbazoles has achieved limited clinical success, with only one therapeutic agent currently approved.

Unlike indolocarbazoles—which predominantly inhibit polymerases/DNA or ATP-binding sites of multiple protein kinases—fascaplysin combines DNA intercalation-induced damage with selective, though relatively weak, inhibition of cyclin-dependent kinases. Its primary mechanism appears to involve induction of metabolic stress, sensed by cellular systems and translated to a protective response comprising: (i) cessation of proliferation-related processes, (ii) activation of autophagy, and (iii) induction of apoptosis. Despite these advances, it must be acknowledged that the mechanisms of action of fascaplysin remain incompletely understood. To study these mechanisms, it is advisable to employ new methodologies, such as PROTAC probe technology and proteomic methods. PROTAC (PROteolysis Targeting Chimeras) methods utilize heterobifunctional molecules that link the protein to be eliminated to E3 ubiquitin ligase. This makes it possible to remove mutant proteins from cells that lead to tumor growth, neurodegeneration, metabolic abnormalities, and other processes, thereby determining the precise molecular mechanisms of action of highly active compounds [154].

Nevertheless, lead compounds derived from fascaplysin already exhibit high efficacy against major medical challenges, including Alzheimer’s disease, solid tumors, and severe infections caused by multidrug-resistant bacteria. Further optimization—focused on pharmacokinetics, delivery strategies, and treatment regimens—is essential to fully exploit the therapeutic and biotechnological potential of fascaplysin.

## Figures and Tables

**Table 1:** Comparison of the pharmacokinetic parameters of fascaplysin, 9-methylfascaplysin (**40**) and doxorubicin.

Pharmacokinetic Parameter	Dox, i.v.	Dox, Oral	Fas, i.v.	Fas, Oral	40, i.v.	40, Oral
Dose, mg/kg	10	10	3	15	5	30
C_max_, µg/L	24,700 ± 14,200	90 ± 10	413.6 ± 39.5	65.2 ± 13.1	1711.7 ± 941.8	193.4 ± 83.5
C_last_, µg/L	120 ± 30	80 ± 10	—	—	12.6 ± 1.0	35.2 ± 9.4
T_max_, h	—	—	0.083	0.25	0.06 ± 0.05	2.7 ± 2.9
MRT_0–t_, h	—	—	2.7 ± 0.4	9.3 ± 0.3	3.9 ± 0.8	9.8 ± 0.4
MRT_0–∞_, h	—	—	9.7 ± 3.8	23.3 ± 9.2	5.6 ± 1.7	19.9 ± 10.0
k_el_, 1/h	0.16 ± 0.02	—	—	—	0.1 ± 0.06	0.06 ± 0.02
T_1/2_, h	4.69 ± 0.8	—	—	—	6.8 ± 3.4	13.4 ± 7.4
AUC_0–t_, µg·h/L	3970 ± 700	330 ± 40	359.5 ± 15.4	358.0 ± 81.9	2321.8 ± 613.5	1960.8 ± 297.9
AUC_0–∞_, µg·h/L	4790 ± 1830	—	412.3 ± 43.6	561.5 ± 124.3	2442.4 ± 619.0	2685.0 ± 535.1
V_d_, L/kg	6.35 ± 2.11	—	870.6	118.3 ± 50.4	21.5908 ± 15.1092	209.7831 ± 81.1215
CL_total_, L/h/kg	2.63 ± 0.39	—	36.7	6.2 ± 3.2	2.1724 ± 0.6013	11.51646 ± 2.1548
F, %	—	8.57 ± 0.71	—	27.2 ± 4.7	—	18.3

## Data Availability

No new data were created or analyzed in this study. Data sharing is not applicable to this study.

## Supplementary Materials

The following supporting information can be downloaded at: https://www.mdpi.com/article/10.3390/md24010018/s1, Table S1: Diversity of natural indolo[2,3-*a*]carbazoles, Table S2: Existing approaches to the synthesis of fascaplysin and its derivatives.
