# Supplementary material for: Can Fascaplysins Be Considered Analogs of Indolo[2,3-a]pyrrolo[3,4-c]carbazoles? Comparison of Biosynthesis, Biological Activity and Therapeutic Potential
Source: Mar Drugs. 2025 Dec 29;24(1):18. doi: 10.3390/md24010018 (PMC12842986; doi:10.3390/md24010018)
Supplement: Supplementary file 1 [file marinedrugs-24-00018-s001.zip › marinedrugs-3996572-supplementary.pdf]

Supplementary Materials

Can Fascaplysins be Considered Analogs of Indolo[2,3-*a*]pyrrolo[3,4-*c*]carbazoles? Comparison of Biosynthesis, Biological Activity and Therapeutic Potential

Maxim E. Zhidkov [1,*], Aleksandr M. Popov [2], Olga A. Soldatkina [1], Oleg A. Tryapkin [1] and Lyubov N. Kharchenko [1]

[1] Department of Chemistry and Materials, Institute of High Technologies and Advanced Materials, FEFU Campus, Far Eastern Federal University, Ajax Bay 10, Russky Island, Vladivostok 690922, Russia; burnevskaia.oa@dvfu.ru (O.A.S.); triapkin_oa@dvfu.ru (O.A.T.); kharchenko.ln@dvfu.ru (L.N.K.)

[2] Departments of Biotechnology and Marine Natural Compounds Chemistry, G.B. Elyakov Pacific Institute of Bioorganic Chemistry, Far Eastern Branch of The Russian Academy of Sciences, Vladivostok 690922, Russia; popovam@piboc.dvo.ru

* Correspondence: zhidkov.me@dvfu.ru

Contents

Table S1 Diversity of natural indolo[2,3-*a*]carbazoles 3-31

Table S2 Existing approaches to the synthesis of fascaplysin and its derivatives 32-37

Diversity of natural indolo[2,3-a]carbazoles

№	Compound	Structure	Organism	Reference
		Derivatives of staurosporine		
1	staurosporine	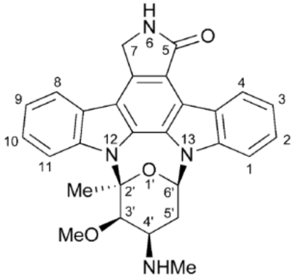	*Streptomyces actuosus*	Morioka, H.; Ishihara, M.; Shibai, H.; Suzuki, T. Agric. Biol. Chem. 1985, 49, 1959.
			Streptomyces sp. M-193	Oka, S.; Kodama, M.; Takeda, H.; Tomizuka, N.; Suzuki, H. Agric. Biol. Chem. 1986, 50, 2723.
			Eudistoma toealensis and their predators *Pseudoceros* sp.	Schupp, P.; Eder, C.; Proksch, P.; Wray, V.; Schneider, B.; Herderich, M.; Paul, V. J. Nat. Prod. 1999, 62, 959.
			Micromonospora sp. L-31CLCO-002	Cañedo Hernández, L. M.; De la Fuente Blanco, J. A.; Pérez Baz, J.; Fernández Puentes, J. L.; Romero Millán, F.; Espliego Vázquez, F.; Fernández-Chimeno, R. I.; García Gravalos, D. J. Antibiot. 2000, 53, 895.
			Streptomyces sp.	Wu, S. J.; Fotso, S.; Li, F.; Qin, S.; Kelter, G.; Fiebig, H. H.; Laatsch, H. J. Antibiot. 2006, 59, 331.
			Streptomyces sp. C-71799	Tanida, S.; Takizawa, M.; Takahashi, T.; Tsubotani, S.; Harada, S. J. Antibiot. 1989, 42, 1619.
				Tsubotani, S.; Tanida, S.; Harada, S. Tetrahedron 1991, 47, 3565.
			Streptomyces sp. AB 1869R359	McAlpine, J.; Karwowski, J.; Jackson, M.; Mullally, M.; Hochlowski, J.; Premachandran, U.; Burres, N. J. Antibiot. 1994, 47, 281.
			Streptomyces sp. N96C-47	Williams, D. E.; Bernan, V. S.; Ritacco, F. V.; Maiese, W. M.; Greenstein, M.; Andersen, R. J. Tetrahedron Lett. 1999, 40, 7171.
			Streptomyces sp. RK-286	Osada, H.; Satake, M.; Koshino, H.; Onose, R.; Isono, K. J. Antibiot. 1992, 45, 278.
			Streptomyces sp. ICN19	Iniyan, A. M.; Sudarman, E.; Wink, J.; Kannan, R. R.; Vincent, S. G. P. J. Antibiot. 2019, 72, 99.
2	Tjipanazole J	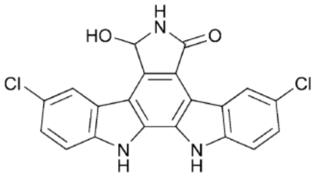	*Tolypothrix* DB-1-1	Bonjouklian, R.; Smitka, T. A.; Doolin, L. E.; Molloy, R. M.; Debono, M.; Shaffer, S. A.; Moore, R. E.; Stewart, J. B.; Patterson, G. M. L. Tetrahedron 1991, 47, 7739.
			Fischerella ambigua	T. Chilczuk, T. F. Schäberle, S. Vahdati, et al. Chembiochem: a European Journal of Chemical Biology 21 (2020): 2170–2177.
3	Tjipanazole K	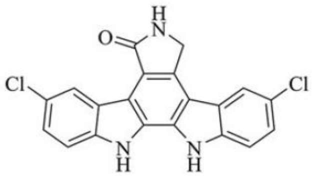	*Fischerella ambigua*	T. Chilczuk, T. F. Schäberle, S. Vahdati, et al. Chembiochem: a European Journal of Chemical Biology 21 (2020): 2170–2177.
4	Tjipanazole L	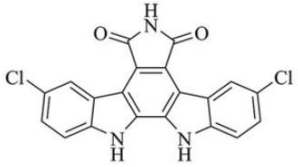	*Fischerella ambigua*	T. Chilczuk, T. F. Schäberle, S. Vahdati, et al. Chembiochem: a European Journal of Chemical Biology 21 (2020): 2170–2177.

5	Tjipanazole M	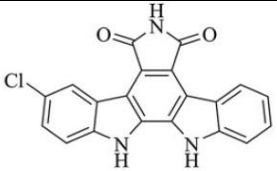	*Fischerella ambigua*	T. Chilczuk, T. F. Schäberle, S. Vahdati, et al. Chembiochem: a European Journal of Chemical Biology 21 (2020): 2170–2177.
6	6-Hydroxy-9'-methoxystaurosporinone	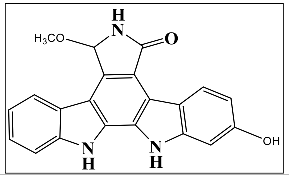	*Perichaena chrysosperma*	Shintani, A.; Toume, K.; Rifai, Y.; Arai, M. A.; Ishibashi, M. J. Nat. Prod. 2010, 73, 1711.
7	6,9'-Dihydroxystaurosporinone	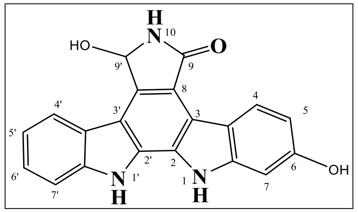	*Arcyria cinerea*	Shintani, A.; Toume, K.; Rifai, Y.; Arai, M. A.; Ishibashi, M. J. Nat. Prod. 2010, 73, 1711.
8	K-252c (staurosporinone)	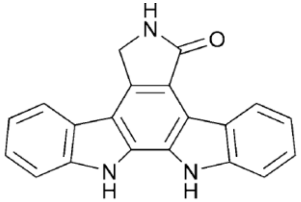	*Nocardiopsis* sp. K-290	Akanishi, S.; Matsuda, Y.; Iwahashi, K.; Kase, H. J. Antibiot. 1986, 39, 1066.
				Yasuzawa, T.; Iida, T.; Yoshida, M.; Hirayama, N.; Takahashi, M.; Shirahata, K.; Sano, H. J. Antibiot. 1986, 39, 1072.
			Lycogala epidendrum	Hosoya, T.; Yamamoto, Y.; Uehara, Y.; Hayashi, M.; Komiyama, K.; Ishibashi, M. Bioorg. Med. Chem. Lett. 2005, 15, 2776.
			Eudistoma sp.	Horton, P. A.; Longley, R. E.; McConnell, O. J.; Ballas, L. M. Experientia 1994, 50, 843.
			Eudistoma toealensis and their predators *Pseudoceros* sp.	Schupp, P.; Eder, C.; Proksch, P.; Wray, V.; Schneider, B.; Herderich, M.; Paul, V. J. Nat. Prod. 1999, 62, 959.
			Streptomyces longisporoflavus R-19	Cai, Y.; Fredenhagen, A.; Hug, P.; Meyer, T.; Peter, H. H. J. Antibiot. 1996, 49, 519.
			Streptomyces sp. Strain 196	Kumar, P.; Kundu, A.; Kumar, M.; Solanki, R.; Kapur, M. K. Microbiol. Res. 2019, 229, 126312.
9	6-Isopropoxymethyl-K-252c	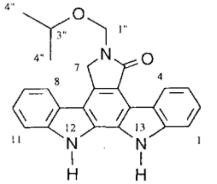	*Streptomyces longisporoflavus* R-19	Cai, Y.; Fredenhagen, A.; Hug, P.; Meyer, T.; Peter, H. H. J. Antibiot. 1996, 49, 519.

10	6-Hydroxystaurosporinone (2-hydroxystaurosporinone)	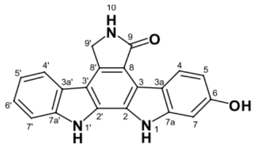	*Lycogala epidendrum*	Hosoya, T.; Yamamoto, Y.; Uehara, Y.; Hayashi, M.; Komiyama, K.; Ishibashi, M. Bioorg. Med. Chem. Lett. 2005, 15, 2776.
			Perichaena chrysosperma	Shintani, A.; Toume, K.; Rifai, Y.; Arai, M. A.; Ishibashi, M. J. Nat. Prod. 2010, 73, 1711.
11	TAN-999	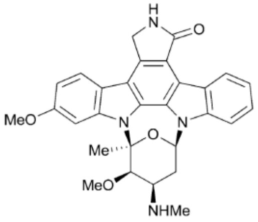	*Nocardiopsis dassonvillei* C-71425	Tanida, S.; Takizawa, M.; Takahashi, T.; Tsubotani, S.; Harada, S. J. Antibiot. 1989, 42, 1619.
				Tsubotani, S.; Tanida, S.; Harada, S. Tetrahedron 1991, 47, 3565.
			Streptomyces sp. C-71799	Tanida, S.; Takizawa, M.; Takahashi, T.; Tsubotani, S.; Harada, S. J. Antibiot. 1989, 42, 1619.
				Tsubotani, S.; Tanida, S.; Harada, S. Tetrahedron 1991, 47, 3565.
12	O-Demethylstaurosporine (3'-demethoxy-3'-hydroxystaurosporine, CGP 58 546)	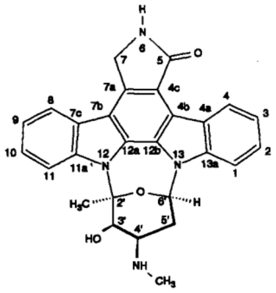	mutant (block of the last step in the biosynthetic pathway) *Streptomyces longisporoflavus* R 19	Hoehn, P.; Ghisalba, O.; Moerker, T.; Peter, H. H. J. Antibiot. 1995, 48, 300.
			Eudistoma toealensis and their predators P*seudoceros* sp.	Schupp, P.; Eder, C.; Proksch, P.; Wray, V.; Schneider, B.; Herderich, M.; Paul, V. J. Nat. Prod. 1999, 62, 959.
			Streptomyces sp. N96C-47	Williams, D. E.; Bernan, V. S.; Ritacco, F. V.; Maiese, W. M.; Greenstein, M.; Andersen, R. J. Tetrahedron Lett. 1999, 40, 7171.
13	11-Hydroxystaurosporine	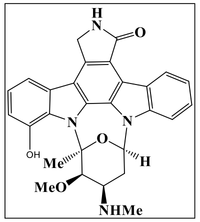	*Eudistoma* sp.	Kinnel, R. B.; Scheuer, P. J. J. Org. Chem. 1992, 57, 6327.
			Eudistoma toealensis and their predators *Pseudoceros* sp.	Schupp, P.; Proksch, P.; Wray, V. J. Nat. Prod. 2002, 65, 295.

14	3,11-Dihydroxystaurosporine	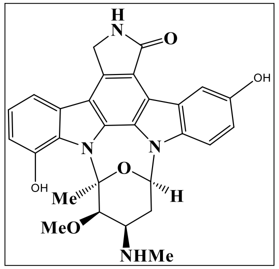	*Eudistoma* sp.	Kinnel, R. B.; Scheuer, P. J. J. Org. Chem. 1992, 57, 6327.
			Coriocella nigra	Cantrell, C. L.; Groweiss, A.; Gustafson, K. R.; Boyd, M. R. Nat. Prod. Lett. 1999, 14, 39.
			Eudistoma toealensis and their predators *Pseudoceros* sp.	Schupp, P.; Eder, C.; Proksch, P.; Wray, V.; Schneider, B.; Herderich, M.; Paul, V. J. Nat. Prod. 1999, 62, 959.
15	11-Hydroxy-4'-N-demethylstaurosporine	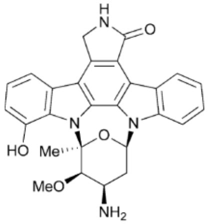	*Coriocella nigra*	Cantrell, C. L.; Groweiss, A.; Gustafson, K. R.; Boyd, M. R. Nat. Prod. Lett. 1999, 14, 39.
			Eudistoma toealensis and their predators *Pseudoceros* sp.	Schupp, P.; Eder, C.; Proksch, P.; Wray, V.; Schneider, B.; Herderich, M.; Paul, V. J. Nat. Prod. 1999, 62, 959.
16	3-Hydroxystaurosporine	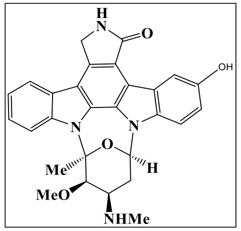	*Eudistoma toealensis* and their predators *Pseudoceros* sp.	Schupp, P.; Eder, C.; Proksch, P.; Wray, V.; Schneider, B.; Herderich, M.; Paul, V. J. Nat. Prod. 1999, 62, 959.
17	3-Hydroxy-3'-O-demethylstaurosporine (3-hydroxy-3'-demethoxy-3'-hydroxystaurosporine)	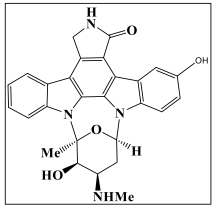	*Eudistoma toealensis* and their predators *Pseudoceros* sp.	Schupp, P.; Eder, C.; Proksch, P.; Wray, V.; Schneider, B.; Herderich, M.; Paul, V. J. Nat. Prod. 1999, 62, 959.
18	4'-N-Demethylstaurosporine	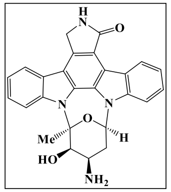	*Eudistoma toealensis* and their predators *Pseudoceros* sp.	Schupp, P.; Eder, C.; Proksch, P.; Wray, V.; Schneider, B.; Herderich, M.; Paul, V. J. Nat. Prod. 1999, 62, 959.

19	5'-Hydroxy-4'-N-methylstaurosporine	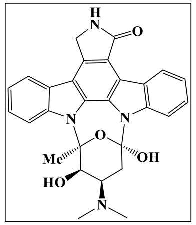	*Micromonospora* sp. L-31CLCO-002	Cañedo Hernández, L. M.; De la Fuente Blanco, J. A.; Pérez Baz, J.; Fernández Puentes, J. L.; Romero Millán, F.; Espliego Vázquez, F.; Fernández-Chimeno, R. I.; García Gravalos, D. J. Antibiot. 2000, 53, 895.
20	5'-Hydroxystaurosporine	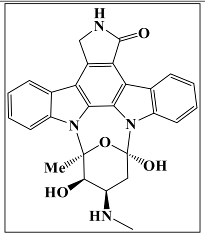	*Micromonospora* sp. L-31CLCO-002	Cañedo Hernández, L. M.; De la Fuente Blanco, J. A.; Pérez Baz, J.; Fernández Puentes, J. L.; Romero Millán, F.; Espliego Vázquez, F.; Fernández-Chimeno, R. I.; García Gravalos, D. J. Antibiot. 2000, 53, 895.
21	4'-N-Methylstaurosporine	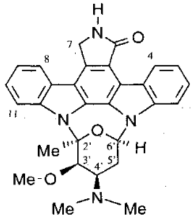	*Streptomyces longisporoflavus* R-19	Cai, Y.; Fredenhagen, A.; Hug, P.; Meyer, T.; Peter, H. H. J. Antibiot. 1996, 49, 1060-1062.
			Eudistoma toealensis and their predators *Pseudoceros* sp.	Schupp, P.; Proksch, P.; Wray, V. J. Nat. Prod. 2002, 65, 295.
22	3-Hydroxy-4'-N-methylstaurosporine	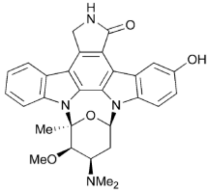	*Eudistoma toealensi* s and their predators *Pseudoceros* sp.	Schupp, P.; Proksch, P.; Wray, V. J. Nat. Prod. 2002, 65, 295.
23	3-Hydroxy-4'-N-demethylstaurosporine	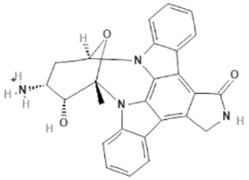	*Eudistoma toealensis* and their predators *Pseudoceros* sp.	Schupp, P.; Proksch, P.; Wray, V. J. Nat. Prod. 2002, 65, 295.

24	3'-O-Demethyl-4'-N-demethylstaurosporine	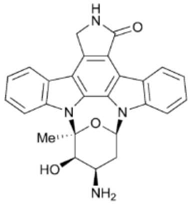	*Eudistoma toealensis* and their predators *Pseudoceros* sp.	Schupp, P.; Proksch, P.; Wray, V. J. Nat. Prod. 2002, 65, 295.
25	N-Formylstaurosporine	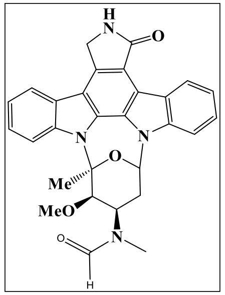	*Streptomyces longsporoflavus* R-19	Cai, Y.; Fredenhagen, A.; Hug, P.; Peter, H. H. J. Antibiot. 1995, 48, 143.
			Streptomyces sp.	Wu, S. J.; Fotso, S.; Li, F.; Qin, S.; Kelter, G.; Fiebig, H. H.; Laatsch, H. J. Antibiot. 2006, 59, 331.
			Streptomyces sp. A22	Cheng, X.; Zhou, B.; Liu, H.; Huo, C.; Ding, W. Nat. Prod. Res. 2018, 32, 2583.
26	4′-N-Demethyl-N-4′-acetylstaurosporine	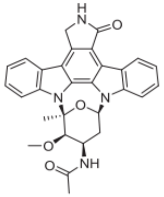	*Streptomyces* sp. A65	B. Zhou, L. L. Qin, W. J. Ding, and Z. J. Ma. Tetrahedron 2018, 74, 726–730.
27	4′-N-Acetyl-staurosporine	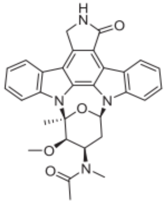	*Streptomyces* sp. A65	B. Zhou, L. L. Qin, W. J. Ding, and Z. J. Ma. Tetrahedron 2018, 74, 726–730.
28	3′-O-Demethyl-4′-N-demethyl-4′-N-acetyl-4′-epi-staurosporine	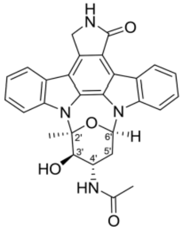	*Streptomyces* sp. DT-A61	Wang, J. N.; Zhang, H. J.; Li, J. Q.; Ding, W. J.; Ma, Z. J. J. Nat. Prod. 2018, 81, 949–956.

29	N-Acetoxymethoxystaurosporine	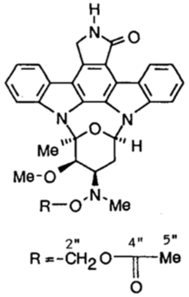	*Streptomyces longsporoflavus* R-19	Cai, Y.; Fredenhagen, A.; Hug, P.; Peter, H. H. J. Antibiot. 1995, 48, 143.
30	N-Hydroxy-4'-N-demethyl-N-formylstaurosporine	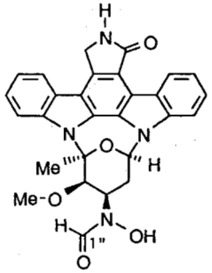	*Streptomyces longsporoflavus* R-19	Cai, Y.; Fredenhagen, A.; Hug, P.; Peter, H. H. J. Antibiot. 1995, 48, 143.
31	4'-Demethylamino-4'-nitrostaurosporine	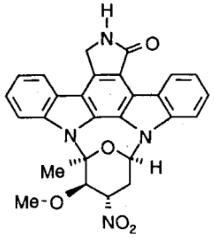	*Streptomyces longsporoflavus* R-19	Cai, Y.; Fredenhagen, A.; Hug, P.; Peter, H. H. J. Antibiot. 1995, 48, 143.
32	N-Carboxamidostaurosporine	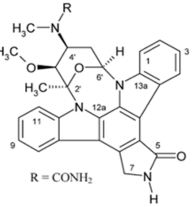	*Streptomyces* sp.	Wu, S. J.; Fotso, S.; Li, F.; Qin, S.; Kelter, G.; Fiebig, H. H.; Laatsch, H. J. Antibiot. 2006, 59, 331.

33	TAN-1030A	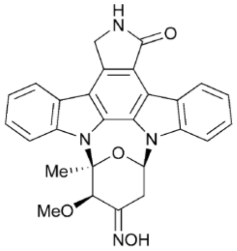	*Streptomyces* sp. C-71799	Tsubotani, S.; Tanida, S.; Harada, S. Tetrahedron 1991, 47, 3565.
			Streptomyces longisporoflavus R19	Cai, Y.; Fredenhagen, A.; Hug, P.; Peter, H. H. J. Antibiot. 1995, 48, 143.
			Streptomyces sp. A22	Cheng, X.; Zhou, B.; Liu, H.; Huo, C.; Ding, W. Nat. Prod. Res. 2018, 32, 2583.
34	6-Methoxymethyl-TAN-1030A	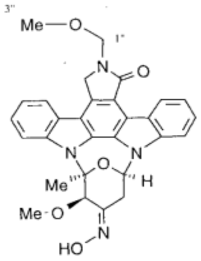	*Streptomyces longisporoflavus* R-19	Cai, Y.; Fredenhagen, A.; Hug, P.; Meyer, T.; Peter, H. H. J. Antibiot. 1996, 49, 519.
35	6-Isopropoxymethyl-TAN-1030A	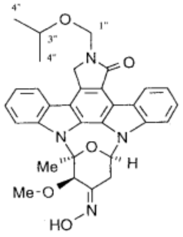	Streptomyces longisporoflavus R-19	Cai, Y.; Fredenhagen, A.; Hug, P.; Meyer, T.; Peter, H. H. J. Antibiot. 1996, 49, 519.
36	4'-Deoxime-4'-oxo-TAN-1030A	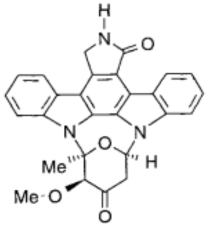	*Streptomyces longisporoflavus* R-19	Cai, Y.; Fredenhagen, A.; Hug, P.; Meyer, T.; Peter, H. H. J. Antibiot. 1996, 49, 519.
37	4'-Deoxime-4'-oxo-3'-epi-TAN1030A	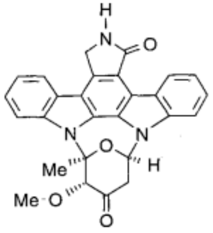	*Streptomyces longisporoflavus* R-19	Cai, Y.; Fredenhagen, A.; Hug, P.; Meyer, T.; Peter, H. H. J. Antibiot. 1996, 49, 519.

38	RK-286C	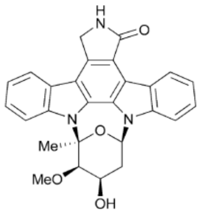	*Streptomyces* sp. RK-286	Osada, H.; Takahashi, H.; Tsunoda, K.; Kusakabe, H.; Isono, K. J. Antibiot. 1990, 43, 163.
			Streptomyces sp. RK-286	Osada, H.; Satake, M.; Koshino, H.; Onose, R.; Isono, K. J. Antibiot. 1992, 45, 278.
39	RK-1409B	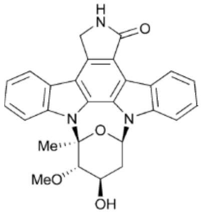	*Streptomyces platensis* subsp. malvinus RK-1409	Koshino, H.; Osada, H.; Amano, S.; Onose, R.; Isono, K. J. Antibiot. 1992, 45, 1428.
			Streptomyces sp. A65	B. Zhou, L. L. Qin, W. J. Ding, and Z. J. Ma. Tetrahedron 2018, 74, 726–730.
40	3′-Demethyl-RK-1409	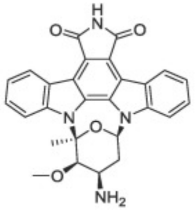	*Streptomyces* sp. NB-A13	Zhou B., Hu Z.J., Zhang H.J., Li J.Q., Ding W.J., Ma Z.J. Bioorg Chem. 2019, 82, 33-40.
41	MLR-52	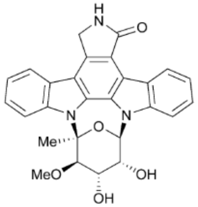	*Streptomyces* sp. AB 1869R359	McAlpine, J.; Karwowski, J.; Jackson, M.; Mullally, M.; Hochlowski, J.; Premachandran, U.; Burres, N. J. Antibiot. 1994, 47, 281.

41			Streptomyces sp. A65	B. Zhou, L. L. Qin, W. J. Ding, and Z. J. Ma. Tetrahedron 2018, 74, 726–730.
42	7-Oxo-MLR-52	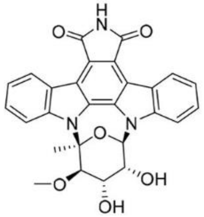	*Streptomyces* sp. NB-A13	Zhou B, Hu ZJ, Zhang HJ, Li JQ, Ding WJ, Ma ZJ. Bioorg Chem., 2019, 33-40.
43	ZHD-0501	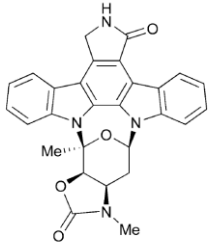	*Actinomadura* sp. 007	Han, X.-X.; Cui, C.-B.; Gu, Q.-Q.; Zhu, W.-M.; Liu, H.-B.; Gu, J.-Y.; Osada, H. Tetrahedron Lett. 2005, 46, 6137.
44	Bmy-41950 (RK-1409, 7-oxostaurosporine)	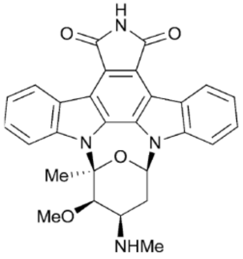	*Streptomyces staurosporeus* R10069	Schroeder, D.; Lam, K. S.; Mattei, J.; Hesler, G. A. EU Patent 388962.
			Streptomyces platensis subsp. malvinus RK-1409	Koshino, H.; Osada, H.; Isono, K. J. Antibiot. 1992, 45, 195.
				Osada, H.; Koshino, H.; Kudo, T.; Onose, R.; Isono, K. J. Antibiot. 1992, 45, 189.
			Streptomyces sanyensis	Cartuche, L.; Reyes-Batlle, M.; Sifaoui, I.; Arberas-Jiménez, I.; Piñero, J. E.; Fernández, J. J.; Lorenzo-Morales, J.; Díaz-Marrero, A. R. Mar. Drugs 2019, 17, 588.
			Streptomyces sp. NB-A13	Zhou B., Hu Z.J., Zhang H.J., Li J.Q., Ding W.J., Ma Z.J. Bioorg Chem., 2019, 33-40.
45	UCN-01	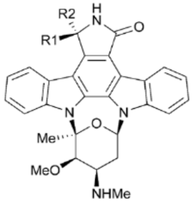 UCN-01 (R1=OH, R2=H)	*Streptomyces* sp. N-71	Takahashi, I.; Kobayashi, E.; Asano, K.; Yoshida, M.; Nakano, H. J. Antibiot. 1987, 40, 1782.

			Streptomyces longisporoflavus R-19	Cai, Y.; Fredenhagen, A.; Hug, P.; Meyer, T.; Peter, H. H. J. Antibiot. 1996, 49, 519.
			Streptomyces sp. N-126	Takahashi, I.; Asano, K.; Kawamoto, I.; Tamaoki, T.; Nakano, H. J. Antibiot. 1989, 42, 564.
				Takahashi, I.; Saitoh, Y.; Yoshida, M.; Sano, H.; Nakano, H.; Morimoto, M.; Tamaoki, T. J. Antibiot. 1989, 42, 571.
46	7-Oxo-TAN-1030A	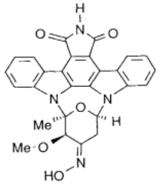	*Streptomyces longisporoflavus* R-19	Cai, Y.; Fredenhagen, A.; Hug, P.; Meyer, T.; Peter, H. H. J. Antibiot. 1996, 49, 519.
47	7-Hydroxy-TAN-1030A	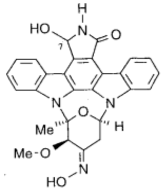	*Streptomyces longisporoflavus* R-19 48	Cai, Y.; Fredenhagen, A.; Hug, P.; Meyer, T.; Peter, H. H. J. Antibiot. 1996, 49, 519.
48	UCN-02	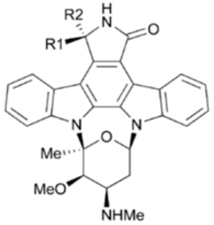 (R1=H, R2=OH)	*Streptomyces* sp. N-126.	Takahashi, I.; Asano, K.; Kawamoto, I.; Tamaoki, T.; Nakano, H. J. Antibiot. 1989, 42, 564.
				Takahashi, I.; Saitoh, Y.; Yoshida, M.; Sano, H.; Nakano, H.; Morimoto, M.; Tamaoki, T. J. Antibiot. 1989, 42, 571.
49	K252d	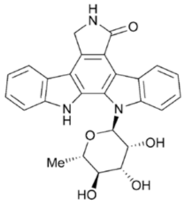	*Nocardiopsis* sp. K-290	Akanishi, S.; Matsuda, Y.; Iwahashi, K.; Kase, H. J. Antibiot. 1986, 39, 1066.
			Streptomyces sp. N96C-47	Williams, D. E.; Bernan, V. S.; Ritacco, F. V.; Maiese, W. M.; Greenstein, M.; Andersen, R. J. Tetrahedron Lett. 1999, 40, 7171.
50	3′-Epi-K252d	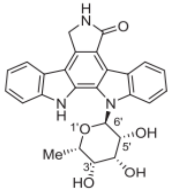	*Streptomyces* sp. A65	B. Zhou, L. L. Qin, W. J. Ding, and Z. J. Ma. Tetrahedron 2018, 74, 726–730.

51	2′,4′-Epi-K252d	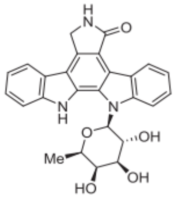	*Streptomyces* sp. A65	B. Zhou, L. L. Qin, W. J. Ding, and Z. J. Ma. Tetrahedron 2018, 74, 726–730.
52	3-Hydroxy-K252d	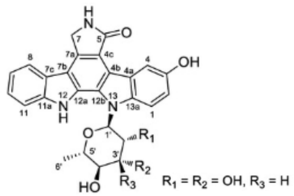	*Feeding Streptomyces* sp. strain OUCMDZ-3118 with 5-hydroxy-l-tryptophan	Wang C., Monger A., Wang L., Fu P., Piyachaturawat P., Chairoungdua A., Zhu W. Mar Drugs., 2018, 16, 168.
53	K252b (H)	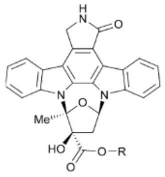	*Nocardiopsis* sp. K-290	Akanishi, S.; Matsuda, Y.; Iwahashi, K.; Kase, H. J. Antibiot. 1986, 39, 1066.
54	7-Oxo-K252b	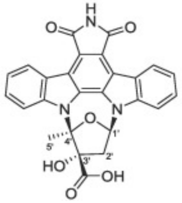	*Streptomyces* sp. NB-A13	Zhou B., Hu Z.J., Zhang H.J., Li J.Q., Ding W.J., Ma Z.J. Bioorg Chem. 2019, 82, 33-40.
55	3′-Epi-5′-methoxy-K252d	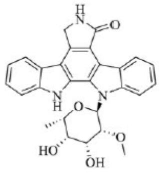	*Streptomyces* sp. NB-A13	Zhou B., Hu Z.J., Zhang H.J., Li J.Q., Ding W.J., Ma Z.J. Bioorg Chem. 2019, 82, 33-40.
56	7-Oxo-k252d	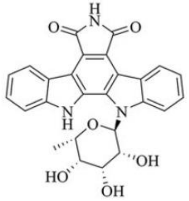	*Streptomyces* sp. NB-A13	Zhou B., Hu Z.J., Zhang H.J., Li J.Q., Ding W.J., Ma Z.J. Bioorg Chem. 2019, 82, 33-40.

57	Holyrine A	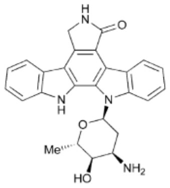	*Streptomyces* sp. N96C-47	Williams, D. E.; Bernan, V. S.; Ritacco, F. V.; Maiese, W. M.; Greenstein, M.; Andersen, R. J. Tetrahedron Lett. 1999, 40, 7171.
58	Holyrine B	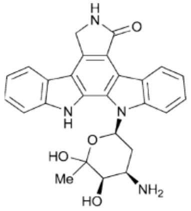	*Streptomyces* sp. N96C-47	Williams, D. E.; Bernan, V. S.; Ritacco, F. V.; Maiese, W. M.; Greenstein, M.; Andersen, R. J. Tetrahedron Lett. 1999, 40, 7171.
59	RK-286D	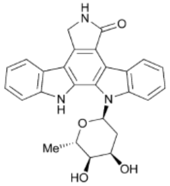	*Streptomyces* sp. RK-286	Osada, H.; Satake, M.; Koshino, H.; Onose, R.; Isono, K. J. Antibiot. 1992, 45, 278.
60	K252a	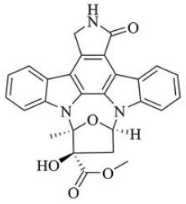	*Streptomyces* sp. A65	B. Zhou, L. L. Qin, W. J. Ding, and Z. J. Ma. Tetrahedron 2018, 74, 726–730.
			Actinomadura sp. SF-2370	Sezaki, M.; Sasaki, T.; Nakazawa, T.; Takeda, U.; Iwata, M.; Watanabe, T.; Koyama, M.; Kai, F.; Shomura, T.; Kojima, M. J. Antibiot. 1985, 38, 1437.
			Streptomyces longisporoflavus R-19	Cai, Y.; Fredenhagen, A.; Hug, P.; Meyer, T.; Peter, H. H. J. Antibiot. 1996, 49, 519.
			Streptomyces sp. NB-A13	Zhou B., Hu Z.J., Zhang H.J., Li J.Q., Ding W.J., Ma Z.J. Bioorg Chem. 2019, 82, 33-40.

61	3'-Methylamino-3'-desoxy-K-252a	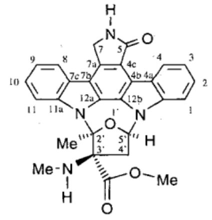	*Streptomyces longisporoflavus* R-19	Cai, Y.; Fredenhagen, A.; Hug, P.; Meyer, T.; Peter, H. H. J. Antibiot. 1996, 49, 1060.
			Streptomyces sp. A22	Cheng, X.; Zhou, B.; Liu, H.; Huo, C.; Ding, W. Nat. Prod. Res. 2018, 32, 2583.
62	Streptocarbazole A	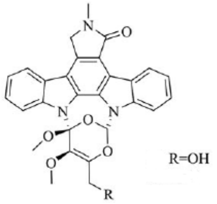	*Streptomyces* sp. FMA	Fu, P.; Yang, C.; Wang, Y.; Liu, P.; Ma, Y.; Xu, L.; Su, M.; Hong, K.; Zhu, W. Org. Lett 2012, 14, 2422.
63	Streptocarbazole B	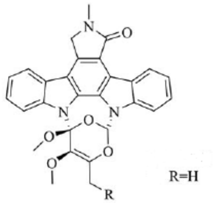	*Streptomyces* sp. FMA	Fu, P.; Yang, C.; Wang, Y.; Liu, P.; Ma, Y.; Xu, L.; Su, M.; Hong, K.; Zhu, W. Org. Lett 2012, 14, 2422.
			Streptomyces sanyensis	Cartuche, L.; Reyes-Batlle, M.; Sifaoui, I.; Arberas-Jiménez, I.; Piñero, J. E.; Fernández, J. J.; Lorenzo-Morales, J.; Díaz-Marrero, A. R. Mar. Drugs 2019, 17, 588.
64	Streptocarbazole C	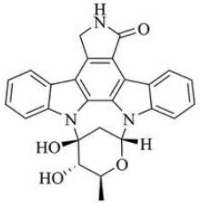	*Streptomyces* sp. A65	B. Zhou, L. L. Qin, W. J. Ding, and Z. J. Ma. Tetrahedron 2018, 74, 726–730.

65	Streptocarbazole D	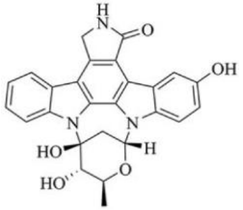	*Streptomyces* sp. DT-A61	Wang, J. N.; Zhang, H. J.; Li, J. Q.; Ding, W. J.; Ma, Z. J. J. Nat. Prod. 2018, 81, 949–956.
66	Streptocarbazole E	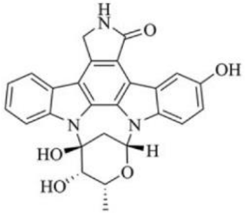	*Streptomyces* sp. DT-A61	Wang, J. N.; Zhang, H. J.; Li, J. Q.; Ding, W. J.; Ma, Z. J. J. Nat. Prod. 2018, 81, 949–956.
67	Streptocarbazole F	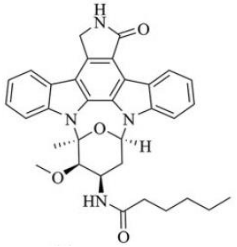	*Streptomyces* sp. OUCMDZ-5380	Cui T., Lin S., Wang Z., Fu .P, Wang C., Zhu W. Front Microbiol. 2022, 13, 957473.
68	Streptocarbazole G	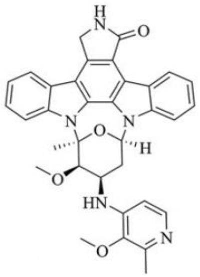	*Streptomyces* sp. OUCMDZ-5380	Cui T., Lin S., Wang Z., Fu .P, Wang C., Zhu W. Front Microbiol. 2022, 13, 957473.
69	Streptocarbazole H	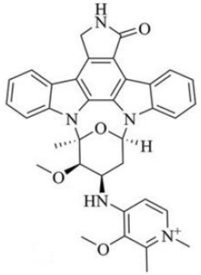	*Streptomyces* sp.OUCMDZ-5380	Cui T., Lin S., Wang Z., Fu .P, Wang C., Zhu W. Front Microbiol. 2022, 13, 957473.

70	K252c	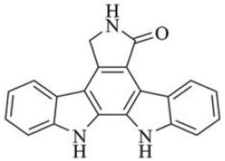	*Streptomyces* sp. A65	B. Zhou, L. L. Qin, W. J. Ding, and Z. J. Ma. Tetrahedron 2018, 74, 726–730.
71	9-Hydroxy-K252c	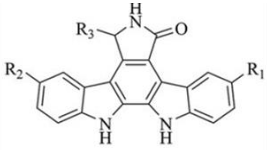 R_1=H, R_2=OH, R_3=H		
72	3-Hydroxy-K252c	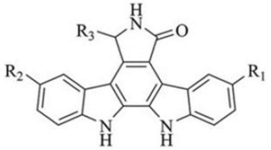 R_1=OH, R_2=R_3=H		
73	3-Hydroxy-7-methoxy-K252c	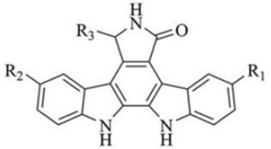 R_1=OH, R_2=H, R_3=OCH_3	*Streptomyces* sp. DT-A61	Wang, J. N.; Zhang, H. J.; Li, J. Q.; Ding, W. J.; Ma, Z. J. J. Nat. Prod. 2018, 81, 949–956.
74	12-N-Methyl-K252c	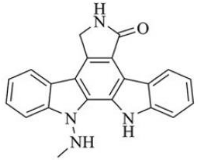	*Streptomyces* sp. A22	Cheng, X.; Zhou, B.; Liu, H.; Huo, C.; Ding, W. Nat. Prod. Res. 2018, 32, 2583.
75	Fradcarbazole A	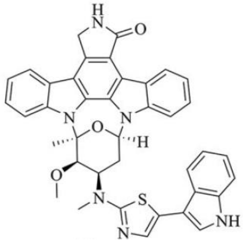	*Streptomyces fradiae* 007M135	Fu, P.; Zhuang, Y.; Wang, Y.; Liu, P.; Qi, X.; Gu, K.; Zhang, D.; Zhu, W. Org. Lett 2012, 14, 6194.

76	Fradcarbazole B	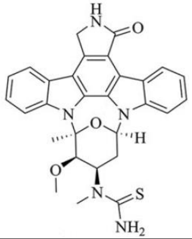	*Streptomyces fradiae* 007M135	Fu, P.; Zhuang, Y.; Wang, Y.; Liu, P.; Qi, X.; Gu, K.; Zhang, D.; Zhu, W. Org. Lett 2012, 14, 6194.
77	Fradcarbazole C	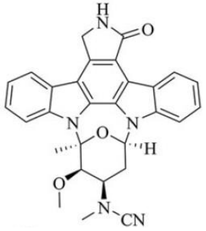	*Streptomyces fradiae* 007M135	Fu, P.; Zhuang, Y.; Wang, Y.; Liu, P.; Qi, X.; Gu, K.; Zhang, D.; Zhu, W. Org. Lett 2012, 14, 6194.
78	3′-Demethylamino-3′-oxostaurosporine	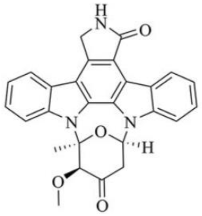	*Streptomyces fradiae* 007M135	Fu, P.; Zhuang, Y.; Wang, Y.; Liu, P.; Qi, X.; Gu, K.; Zhang, D.; Zhu, W. Org. Lett 2012, 14, 6194.
			Streptomyces longisporoflavus	Cai, Y.; Fredenhagen, A.; Hug, P.; Meyer, T.; Peter, H. H. J. Antibiot. 1996, 49, 519.
79	N-Acetyl-3'-epiholyrine A	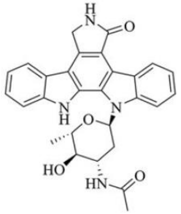	*Streptomyces* sp. A68 and mutant strain R-M1 68	Qin, L.-L.; Zhou, B.; Ding, W.; Ma, Z. Phytochem. Lett. 2018, 23, 46.
80	3'-N-Acetylholyrine A	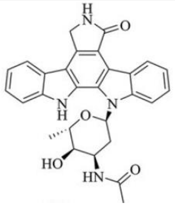	*Streptomyces* sp. A68 and mutant strain R-M1	Qin, L.-L.; Zhou, B.; Ding, W.; Ma, Z. Phytochem. Lett. 2018, 23, 46.

81	9-Hydroxy-3′-N-acetylholyrine A	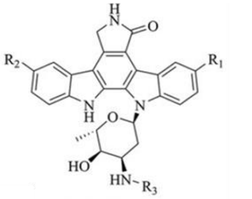 R_1=H, R_2=OH, R_3=$COCH_3$	Streptomyces sp. DT-A61	Wang, J. N.; Zhang, H. J.; Li, J. Q.; Ding, W. J.; Ma, Z. J. J. Nat. Prod. 2018, 81, 949–956.
82	3-Hydroxy-3′-N-acetylholyrine A	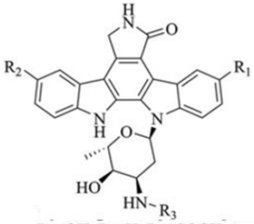 R_1=OH, R_2=H, R_3=$COCH_3$	Streptomyces sp. DT-A61	Wang, J. N.; Zhang, H. J.; Li, J. Q.; Ding, W. J.; Ma, Z. J. J. Nat. Prod. 2018, 81, 949–956.
			Feeding Streptomyces sp. strain OUCMDZ-3118 with 5-hydroxy-l-tryptophan	Wang C., Monger A., Wang L., Fu P., Piyachaturawat P., Chairoungdua A., Zhu W. Mar Drugs. 2018, 16, 168
83	3-Hydroxyholyrine A	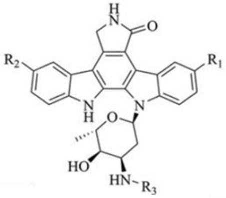 R_1=OH, R_2=R_3=H	Streptomyces sp. DT-A61	Wang, J. N.; Zhang, H. J.; Li, J. Q.; Ding, W. J.; Ma, Z. J. J. Nat. Prod. 2018, 81, 949–956.
			Feeding Streptomyces sp. strain OUCMDZ-3118 with 5-hydroxy-l-tryptophan	Wang C., Monger A., Wang L., Fu P., Piyachaturawat P., Chairoungdua A., Zhu W. Mar Drugs. 2018, 16, 168
84	3'-N-Formylholyrine A	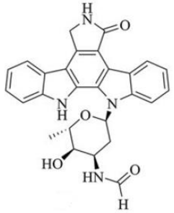	*Streptomyces* sp. A68 and mutant strain R-M1	Qin, L.-L.; Zhou, B.; Ding, W.; Ma, Z. Phytochem. Lett. 2018, 23, 46.
85	7-Oxo-holyrin A	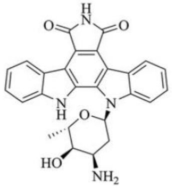	*Streptomyces* sp. NB-A13	Zhou B., Hu Z.J., Zhang H.J., Li J.Q., Ding W.J., Ma Z.J. Bioorg Chem. 2019, 82, 33-40.

86	3′-N-Formyl-7-oxo-holyrin A	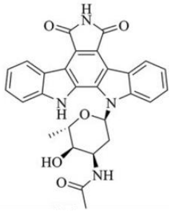	*Streptomyces* sp. NB-A13	Zhou B., Hu Z.J., Zhang H.J., Li J.Q., Ding W.J., Ma Z.J. Bioorg Chem. 2019, 82, 33-40.
87	4′-(Hydroxyl(oxiran-2-yl)methoxy)-holyrine A	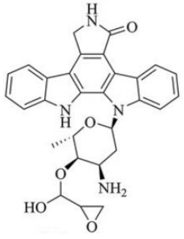	*Streptomyces* sp. NB-A13	Zhou B., Hu Z.J., Zhang H.J., Li J.Q., Ding W.J., Ma Z.J. Bioorg Chem. 2019, 82, 33-40.
88	A61-34G	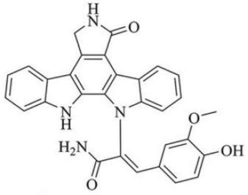	*Streptomyces* sp. NB-A13	J. Wang, W. Jin, X. Zhou, et al., Journal of Medicinal Chemistry 63 (2020): 12978–12991.
89	M1-5E	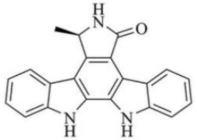	*Streptomyces* sp. NB-A13	Wang J., Jin W., Zhou X., Li J., Xu C., Ma Z., Wang J., Qin L., Zhou B., Ding W., Gao T., Yao H., Chen Z. J Med Chem. 2020, 63, 12978-12991
90	3′-Epi-4′-oxo-staurosporine	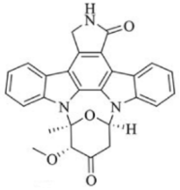	*Streptomyces* sp. A65	B. Zhou, L. L. Qin, W. J. Ding, and Z. J. Ma. Tetrahedron 2018, 74, 726–730.
91	(R)-l-Digitoxosyl-arcyriaflavin A	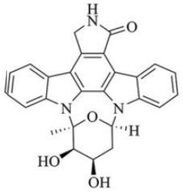	*Streptomyces* sp. A22	Cheng, X.; Zhou, B.; Liu, H.; Huo, C.; Ding, W. Nat. Prod. Res. 2018, 32, 2583.

92	4′-Oxo-staurosporine	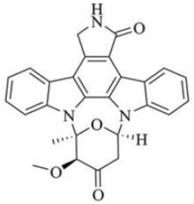	*Streptomyces* sp. A22	Cheng, X.; Zhou, B.; Liu, H.; Huo, C.; Ding, W. Nat. Prod. Res. 2018, 32, 2583.
93	13G-22I	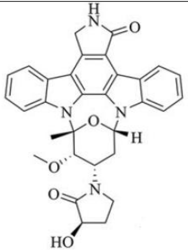	*Streptomyces* sp. SS-13G	Wang J., Jin W., Zhou X., Li J., Xu C., Ma Z., Wang J., Qin L., Zhou B., Ding W., Gao T., Yao H., Chen Z. J Med Chem. 2020, 63, 12978-12991
94	13G-20E	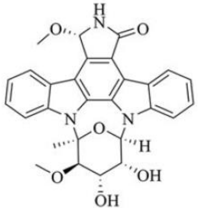	*Streptomyces* sp. SS-13G	Wang J., Jin W., Zhou X., Li J., Xu C., Ma Z., Wang J., Qin L., Zhou B., Ding W., Gao T., Yao H., Chen Z. J Med Chem. 2020, 63, 12978-12991
95	13G-28L	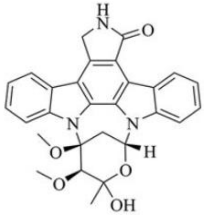	*Streptomyces* sp. SS-13G	Wang J., Jin W., Zhou X., Li J., Xu C., Ma Z., Wang J., Qin L., Zhou B., Ding W., Gao T., Yao H., Chen Z. J Med Chem. 2020, 63, 12978-12991
96	13G-20F	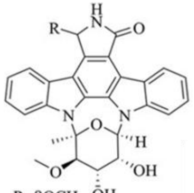 R=βOCH$_3$		

97	13G-22F	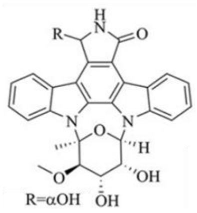	*Streptomyces* sp. SS-13G	Wang J., Jin W., Zhou X., Li J., Xu C., Ma Z., Wang J., Qin L., Zhou B., Ding W., Gao T., Yao H., Chen Z. J Med Chem. 2020, 63, 12978-12991
98	13G-22G	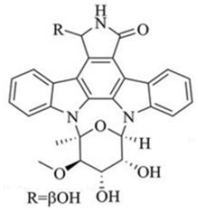		
99	13G-28G	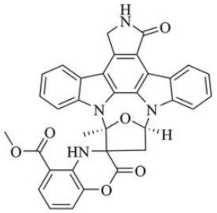	*Streptomyces* sp. SS-13G	Wang J., Jin W., Zhou X., Li J., Xu C., Ma Z., Wang J., Qin L., Zhou B., Ding W., Gao T., Yao H., Chen Z. J Med Chem. 2020, 63, 12978-12991
100	13G-22D	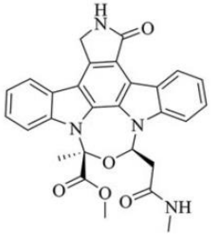	*Streptomyces* sp. SS-13G	Wang J., Jin W., Zhou X., Li J., Xu C., Ma Z., Wang J., Qin L., Zhou B., Ding W., Gao T., Yao H., Chen Z. J Med Chem. 2020, 63, 12978-12991
101	M1-3D	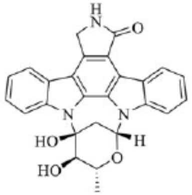	*Streptomyces* sp. TZ-A47	Wang J., Jin W., Zhou X., Li J., Xu C., Ma Z., Wang J., Qin L., Zhou B., Ding W., Gao T., Yao H., Chen Z. J Med Chem. 2020, 63, 12978-12991
**Derivatives of rebeccamycin**				

102	Rebeccamycin	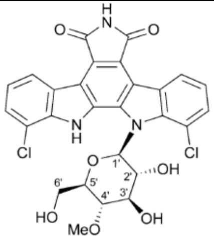	*Lechevalieria aerocolonigenes* (*Nocardia aerocolonigenes* C-38383-RK-2 (ATCC 39243), *Saccharothrix aerocolonigenes*)	D. E. Nettleton, T. W. Doyle, B. Krishnan, G. K. Matsumoto and J. Clardy, Tetrahedron Lett., 1985, 26, 4011–4014.
				J. A. Bush, B. H. Long, J. J. Catino, W. T. Bradner and K. Tomita, J. Antibiot., 1987, 40, 668–678.
103	Arcyriaflavin A	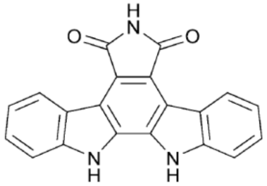	*Arcyria nutans*	Gill, M.; Steglich, W. In Fortschritte der Chemie organischer Naturstoffe. Progress in the Chemistry of Organic Natural Products; Herz, W.; Grisebach, H.; Kirby, G. W.; Tamm, Ch., Eds.; Springer-Verlag: Wien, 1987, Vol. 51, p. 1.
				Steglich, W. Pure Appl. Chem. 1989, 61, 281.
			Lycogala epidendrum	Hosoya, T.; Yamamoto, Y.; Uehara, Y.; Hayashi, M.; Komiyama, K.; Ishibashi, M. Bioorg. Med. Chem. Lett. 2005, 15, 2776.
			Eudistoma sp.	Horton, P. A.; Longley, R. E.; McConnell, O. J.; Ballas, L. M. Experientia 1994, 50, 843.
104	Arcyriaflavin B	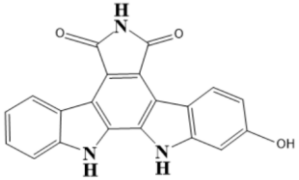	*Arcyria denudata*	Steglich, W. Pure Appl. Chem. 1989, 61, 281.
				Steglich, W. Pure Appl. Chem. 1981, 53, 1233.
				Steglich, W.; Steffan, B.; Kopanski, L.; Eckhardt, G. Angew. Chem., Int. Ed. Engl. 1980, 19, 459.
			Metatrichia vesparium	Kopanski, L.; Li, G.-R.; Besl, H.; Steglich, W. Liebigs Ann. Chem. 1982, 1722.
			Lycogala epidendrum	Hosoya, T.; Yamamoto, Y.; Uehara, Y.; Hayashi, M.; Komiyama, K.; Ishibashi, M. Bioorg. Med. Chem. Lett. 2005, 15, 2776.
			Tubifera casparyi	Nakatani, S.; Naoe, A.; Yamamoto, Y.; Yamauchi, T.; Yamaguchi, N.; Ishibashi, M. Bioorg. Med. Chem. Lett. 2003, 13, 2879.
			Lycogala epidendrum	Hosoya, T.; Yamamoto, Y.; Uehara, Y.; Hayashi, M.; Komiyama, K.; Ishibashi, M. Bioorg. Med. Chem. Lett. 2005, 15, 2776.
			Arcyria denudata	Steglich, W. Pure Appl. Chem. 1989, 61, 281.
				Steglich, W. Pure Appl. Chem. 1981, 53, 1233.
				Steglich, W.; Steffan, B.; Kopanski, L.; Eckhardt, G. Angew. Chem., Int. Ed. Engl. 1980, 19, 459.
			Perichaena chrysosperma	Shintani, A.; Toume, K.; Rifai, Y.; Arai, M. A.; Ishibashi, M. J. Nat. Prod. 2010, 73, 1711.
105	Arcyriaflavin C	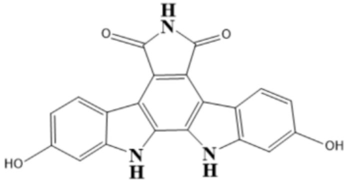	*Metatrichia vesparium*	Kopanski, L.; Li, G.-R.; Besl, H.; Steglich, W. Liebigs Ann. Chem. 1982, 1722.
			Tubifera casparyi	Nakatani, S.; Naoe, A.; Yamamoto, Y.; Yamauchi, T.; Yamaguchi, N.; Ishibashi, M. Bioorg. Med. Chem. Lett. 2003, 13, 2879.

106	Arcyriaflavin D	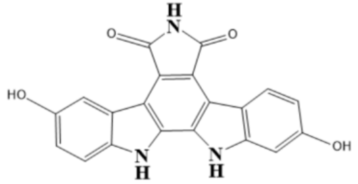	*Dictydiaethalium plumbeum*	Steglich, W. Pure Appl. Chem. 1989, 61, 281.
107	Arcyriaflavin E	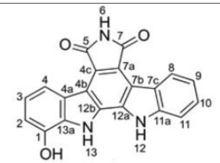	mixed culture of *Tsukamurella pulmonis* and *Streptomyces cinnamoneus* NBRC 13823 22	Hoshino, S.; Zhang, L.; Awakawa, T.; Wakimoto, T.; Onaka, H.; Abe, I. J. Antibiot. 2015, 68, 342.
108	5,6-Dihydroxyarcyriaflavin A	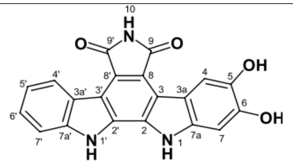	*Lycogala epidendrum*	Hosoya, T.; Yamamoto, Y.; Uehara, Y.; Hayashi, M.; Komiyama, K.; Ishibashi, M. Bioorg. Med. Chem. Lett. 2005, 15, 2776.
109	BE-13793C	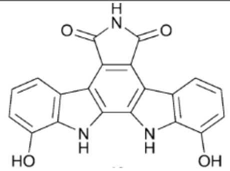	*Streptoverticillium mobaraense* BA13793	Kojiri, K.; Kondo, H.; Yoshinari, T.; Arakawa, H.; Nakajima, S.; Satoh, F.; Kawamura, K.; Okura, A.; Suda, H.; Okanishi, M. J. Antibiot. 1991, 44, 723.
			mixed culture of *Streptomyces* sp. MA37 and *Pseudomonas* sp.	Maglangit, F.; Fang, Q.; Kyeremeh, K.; Sternberg, J. M.; Ebel, R.; Deng, H. Molecules 2020, 25, 256.
110	(+)-indocarbazostatin	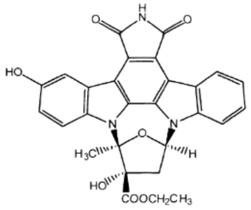		
111	(−)-indocarbazostatin B	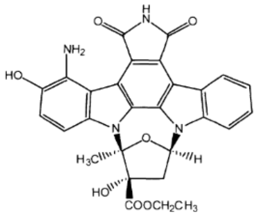		

112	(+)-Indocarbazostatin C	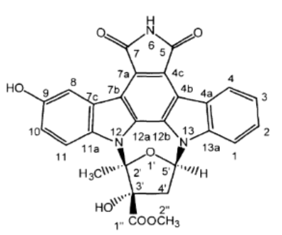	*Streptomyces* sp. MUV-6-83 25	Feng, Y.; Matsuura, N.; Ubukata, M. J. Antibiot. 2004, 57, 627.
113	(−)-indocarbazostatin D	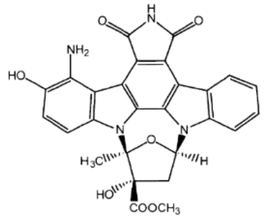		
114	4'-deschlororebeccamycin	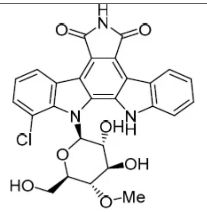	*Saccharothrix aerocolonigenes*	Matson, J. A. US Patent 4,524,145.
115	7-Oxo-3,8,9-trihydroxystaurosporine	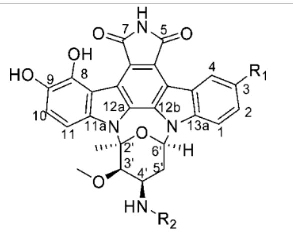 R_1= OH, R_2= Me	*Cystodytes solitus Monniot*	Reyes, F.; Fernández, R.; Rodríguez, A.; Bueno, S.; de Eguilior, C.; Francesch, A.; Cuevas, C. J. Nat. Prod. 2008, 71, 1046.
116	7-Oxo-8,9-dihydroxy-4'-N-demethylstaurosporine	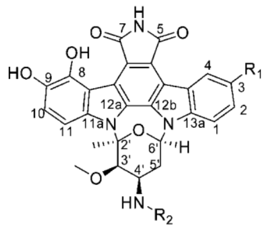 R_1= R_2= H	*Cystodytes solitus Monniot*	Reyes, F.; Fernández, R.; Rodríguez, A.; Bueno, S.; de Eguilior, C.; Francesch, A.; Cuevas, C. J. Nat. Prod. 2008, 71, 1046.

117-120	AT2433-A1, AT2433-A2, AT2433-B1, and AT2433-B2	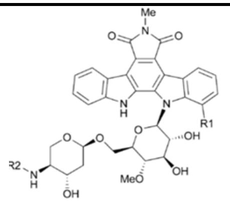<br>AT2433-A1 (R1=Cl, R2=Me)<br>AT2433-A2 (R1=Cl, R2=H)<br>AT2433-B1 (R1=H, R2=Me)<br>AT2433-B2 (R1=R2=H)	*Actinomadura melliaura*	Golik, J.; Doyle, T. W.; Krishnan, B.; Dubay, G.; Matson, J. A. J. Antibiot. 1989, 42, 1784.<br>Horan, A. C.; Golik, J.; Matson, J. A.; Patel, M. G. EU Patent 175284.<br>Matson, J. A.; Claridge, C.; Bush, J. A.; Titus, J.; Bradner, W. T.; Doyle, T. W.; Horan, A. C.; Patel, M. J. Antibiot. 1989, 42, 1547.
121-124	AT2433-A3, AT2433-A4, AT2433-A5, and AT2433-B3	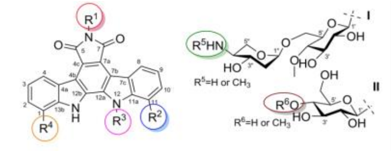<br>$R^1=R^4=H$, $R^2=Cl$, $R^3=I$, $R^5=CH_3$; AT2433-A3<br>$R^1=R^6=CH_3$, $R^4=H$, $R^2=Cl$, $R^3=II$; AT2433-A4<br>$R^1=R^3=R^4=H$, $R^2=Cl$; AT2433-A5<br>$R^1=R^6=CH_3$, $R^2=R^4=H$, $R^3=II$; AT2433-B3	*Actinomadura melliaura*	Shaaban, K. A.; Elshahawi, S. I.; Wang, X.; Horn, J.; Kharel, M. K.; Leggas, M.; Thorson, J. S. J. Nat. Prod. 2015, 78, 1723.
125	9-Methoxyrebeccamycin	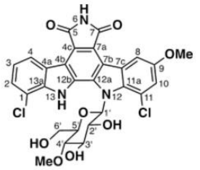	*Pseudonocardia* sp. (isolates derived from anthills of *Apterostigma dentigerum*)	Van Arnam, E. B.; Ruzzini, A. C.; Sit, C. S.; Currie, C. R.; Clardy, J. J. Am. Chem. Soc. 2015, 137, 14272.
126	Loonamycin A	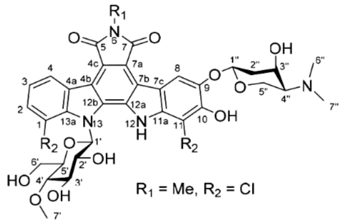	*Nocardiopsis flavescens* NA01583	C. L. Yang, B. Zhang, W. W. Xue, W. Li, Z. F. Xu, J. Shi, Y. Shen, R. H. Jiao, R. X. Tan, H. M. Ge Organic Letters, 2020, 22, 4665-4669 .
127	Loonamycin B	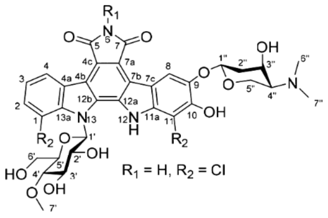	*Nocardiopsis flavescens* NA01583	C. L. Yang, B. Zhang, W. W. Xue, W. Li, Z. F. Xu, J. Shi, Y. Shen, R. H. Jiao, R. X. Tan, H. M. Ge Organic Letters, 2020, 22, 4665-4669 .

128	Loonamycin C	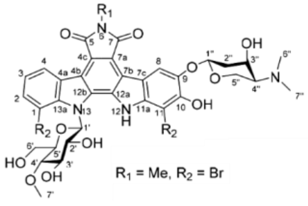	*Nocardiopsis flavescens* NA01583	C. L. Yang, B. Zhang, W. W. Xue, W. Li, Z. F. Xu, J. Shi, Y. Shen, R. H. Jiao, R. X. Tan, H. M. Ge Organic Letters, 2020, 22, 4665-4669 .
129-130	A47-17E and A47-17C	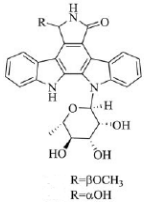	*Streptomyces* sp. TZ-A47	Wang J., Jin W., Zhou X., Li J., Xu C., Ma Z., Wang J., Qin L., Zhou B., Ding W., Gao T., Yao H., Chen Z. J Med Chem. 2020, 63, 12978-12991
131	13G-31G	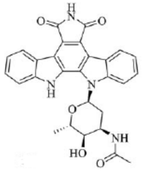	*Streptomyces* sp. SS-13G	Wang J., Jin W., Zhou X., Li J., Xu C., Ma Z., Wang J., Qin L., Zhou B., Ding W., Gao T., Yao H., Chen Z. J Med Chem. 2020, 63, 12978-12991
**Indolo[2,3-*a*]carbazoles without pyrrole ring**				
132	6-Methoxy-11-methylindolo[2,3-a]carbazole-5-carbonitrile	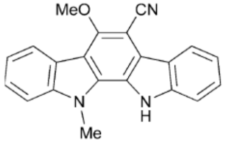	*Nostoc sphaericum* EX-5-1	
133	6-Methoxyindolo[2,3-a]carbazole-5-carbonitrile (H)	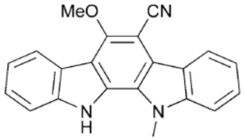	*Nostoc sphaericum* EX-5-1	
134	Tjipanazole A1	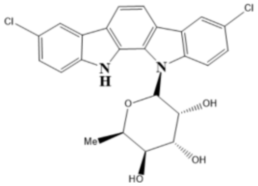		

135	Tjipanazole A2	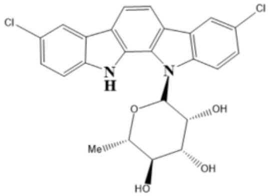	*Tolypothrix tjipanasensis* DB-1-1, *Fischerella ambigua*	Knübel, G.; Larsen, L. K.; Moore, R. E.; Levine, I. A.; Patterson, G. M. J. Antibiot. 1990, 43, 1236. Bonjouklian, R.; Smitka, T. A.; Doolin, L. E.; Molloy, R. M.; Debono, M.; Shaffer, S. A.; Moore, R. E.; Stewart, J. B.; Patterson, G. M. L. Tetrahedron 1991, 47, 7739. Falch, B. S.; Koenig, G. M.; Wright, A. D.; Sticher, O.; Ruegger, H.; Bernardinelli, G. J. Org. Chem. 1993, 58, 6570. Wright, A. D.; Papendorf, O.; König, G. M. J. Nat. Prod. 2005, 68(3), 459.
136	Tjipanazole B	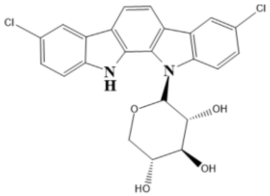		
137	Tjipanazole C1	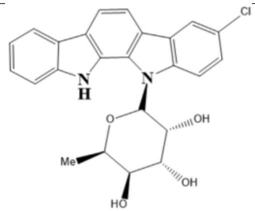		
138	Tjipanazole C2	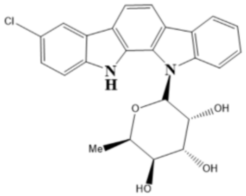		
139	Tjipanazole C3	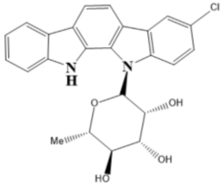		
140	Tjipanazole C4	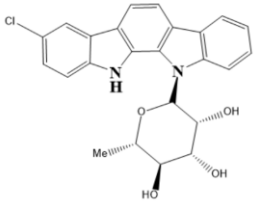		

141	Tjipanazole D	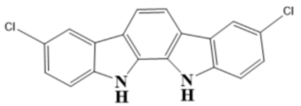		
142	Tjipanazole F1	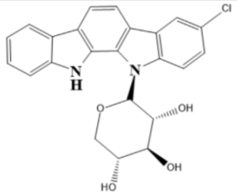		
143	Tjipanazole F2	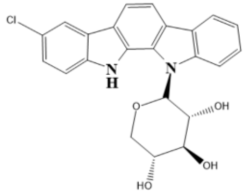		
144	Tjipanazole G1	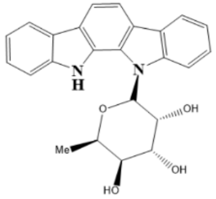		
145	Tjipanazole G2	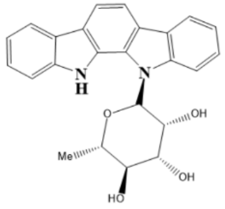		
146	Tjipanazole E	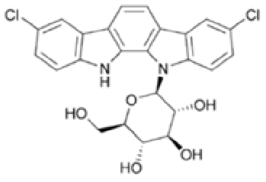		

147	Tjipanazole I	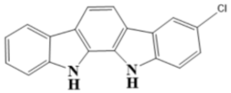		

Existing approaches to the synthesis of fascaplysin and its derivatives

Synthesis scheme	***Reference***
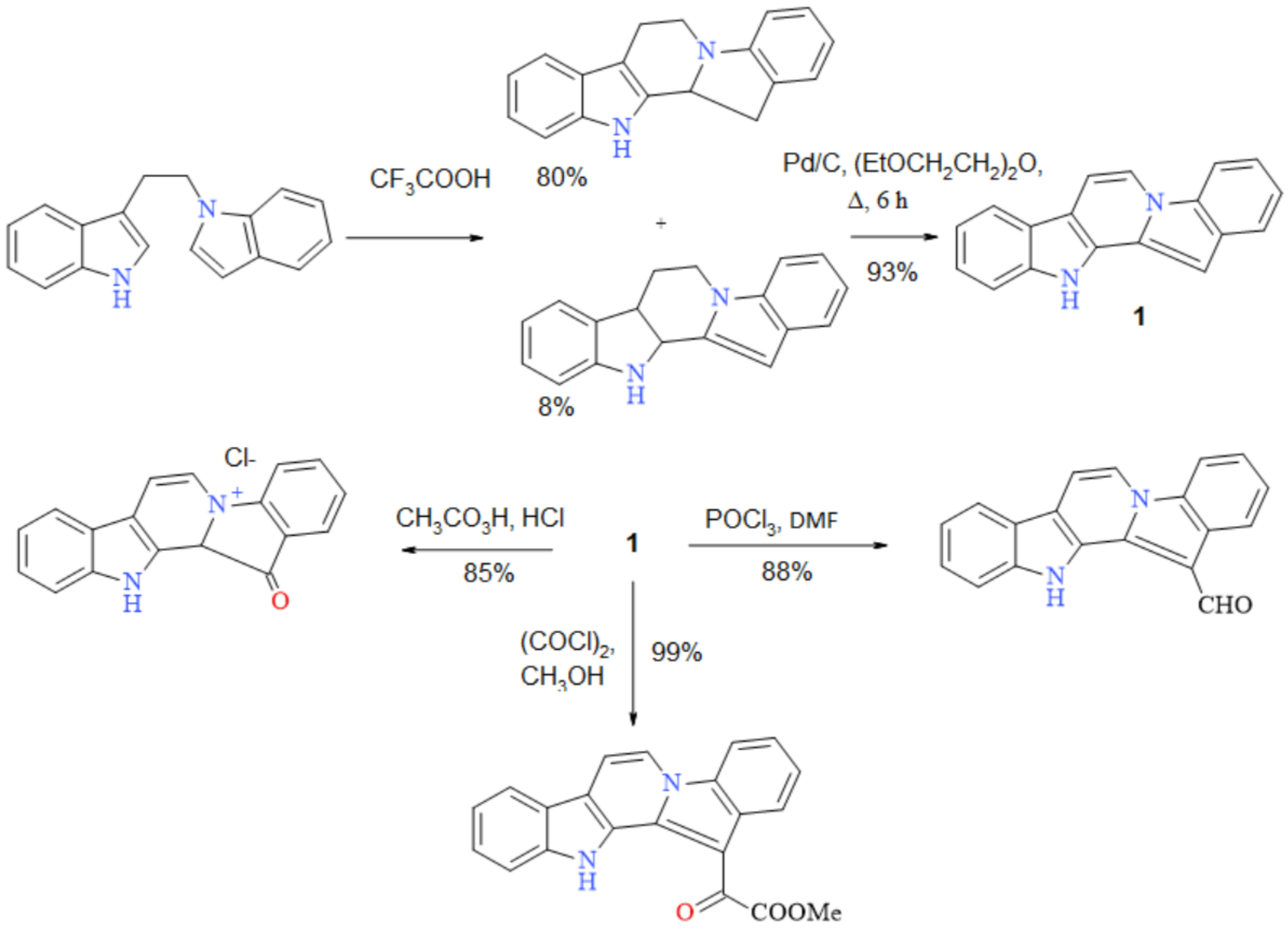	Gribble, G.W.; Pelcman, B. Total syntheses of the marine sponge pigments fascaplysin and homofascaplysin B and C. J. Org. Chem. 1992, 57, 3636. https://doi.org/10.1021/jo00039a024

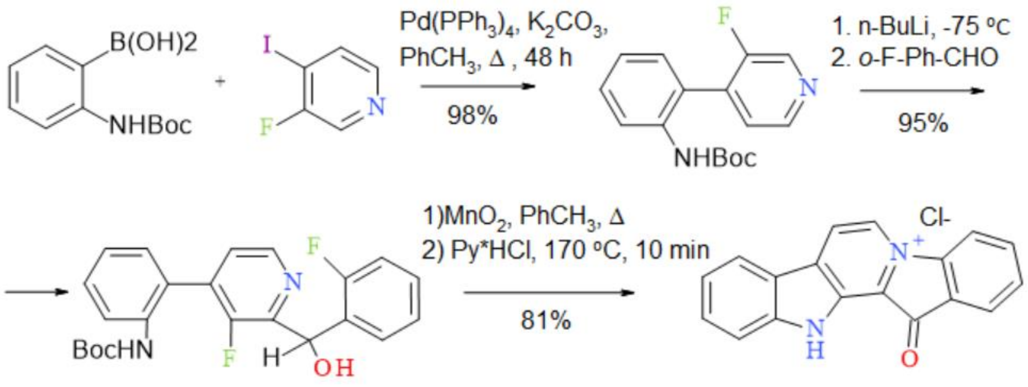

Rocca, P.; Marsais, F.; Godard, A.; Quéguiner, G. A short synthesis of the antimicrobial marine sponge pigment fascaplysin. Tetrahedron Lett. 1993, 34, 7917. https://doi.org/10.1016/s0040-4039(00)61510-1.

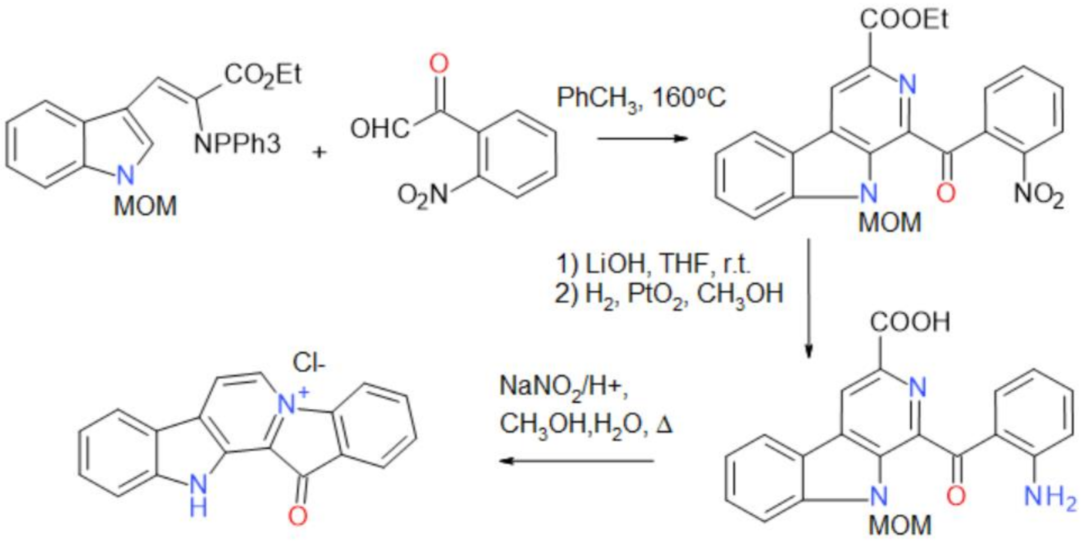

Molina, P.; Fresneda, P.M.; García-Zafra, S.; Almendros, P. Iminophosphorane-mediated syntheses of the fascaplysin alkaloid of marine origin and nitramarine. Tetrahedron Lett. 1994, 35, 8851. https://doi.org/10.1016/s0040-4039(00)78515-7.

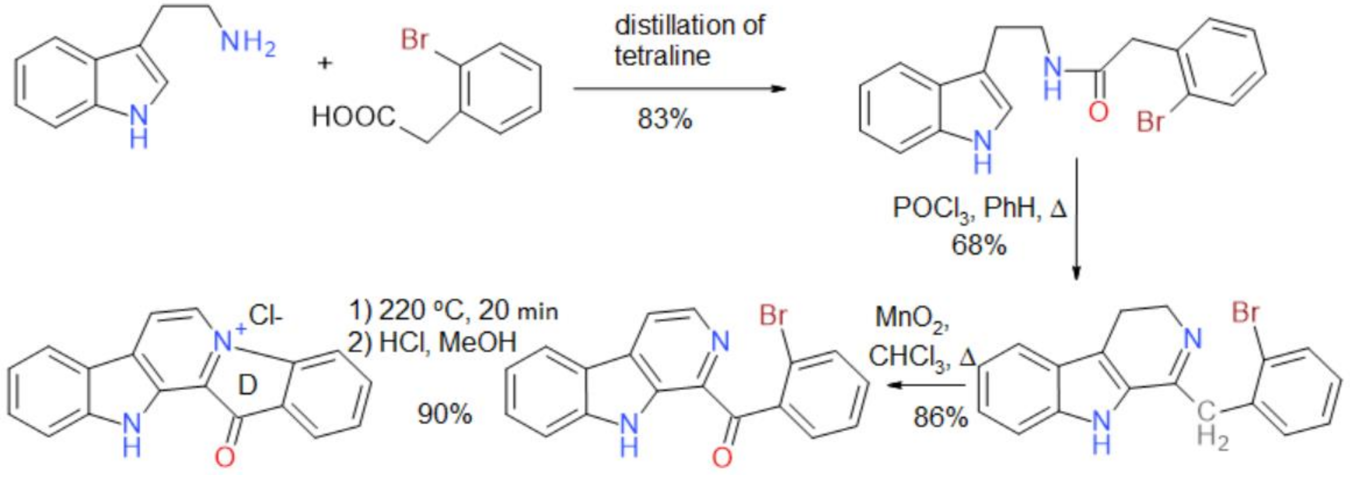

Radchenko, O.S.; Novikov, V.L.; Elyakov, G.B. A simple and practical approach to the synthesis of the marine sponge pigment fascaplysin and related compounds. Tetrahedron Lett. 1997, 38, 5339. https://doi.org/10.1016/s0040-4039(97)01167-2.

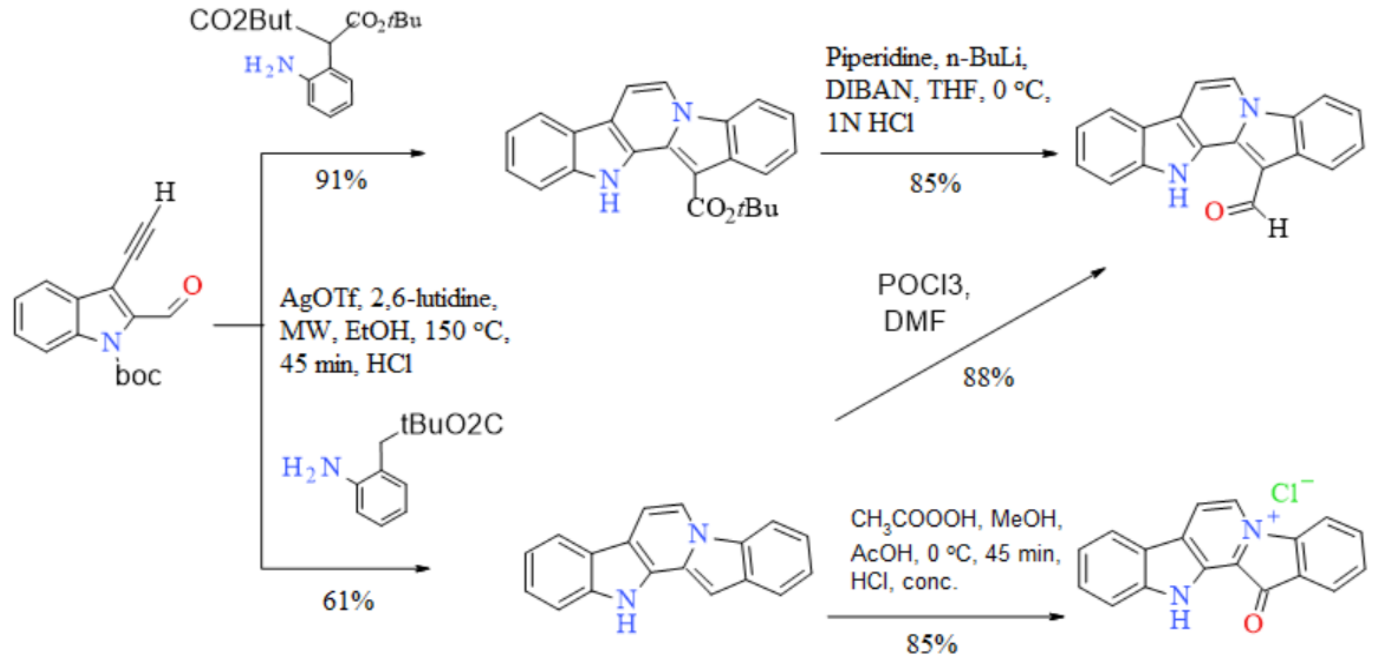

Waldmann, H.; Eberhardt, L.; Wittstein, K.; Kumar, K. Silver catalyzed cascade synthesis of alkaloid ring systems: Concise total synthesis of fascaplysin, homofascaplysin C and analogues. Chem. Commun. 2010, 46, 4622. https://doi.org/10.1039/c001350a.

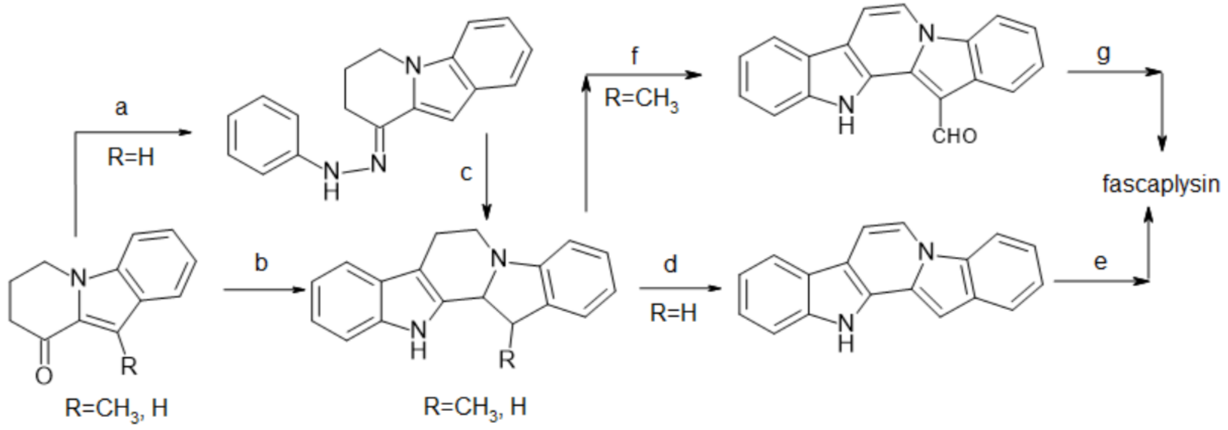

Reagents and conditions: (a). Ph-NHNH$_2$, HCl, EtOH, 2 h, 78%; (b). Ph-NH-NH$_2$, HCl, AcOH, 120 °C, 4 h, 91%; (c). TsOH, PhH, 80 °C, 20 min, 60%; (d). Pd/C, $(EtOCH_2CH_2)_2O$, 180 °C, 6 h, 75%; (e) CH_3CO_3H, MeOH, 0 °C, 45 min, 85%; (f). DDQ, 1,4-dioxane, 100 °C, 2 h, 50%; (g). *m*-CPBA, EtOAc, 24 h, 67%.

Zhidkov, M.E.; Baranova, O.V.; Kravchenko, N.S.; Dubovitskii, S.V. A new method for the synthesis of the marine alkaloid fascaplysin. Tetrahedron Lett. 2010, 51, 6498. https://doi.org/10.1016/j.tetlet.2010.09.120.

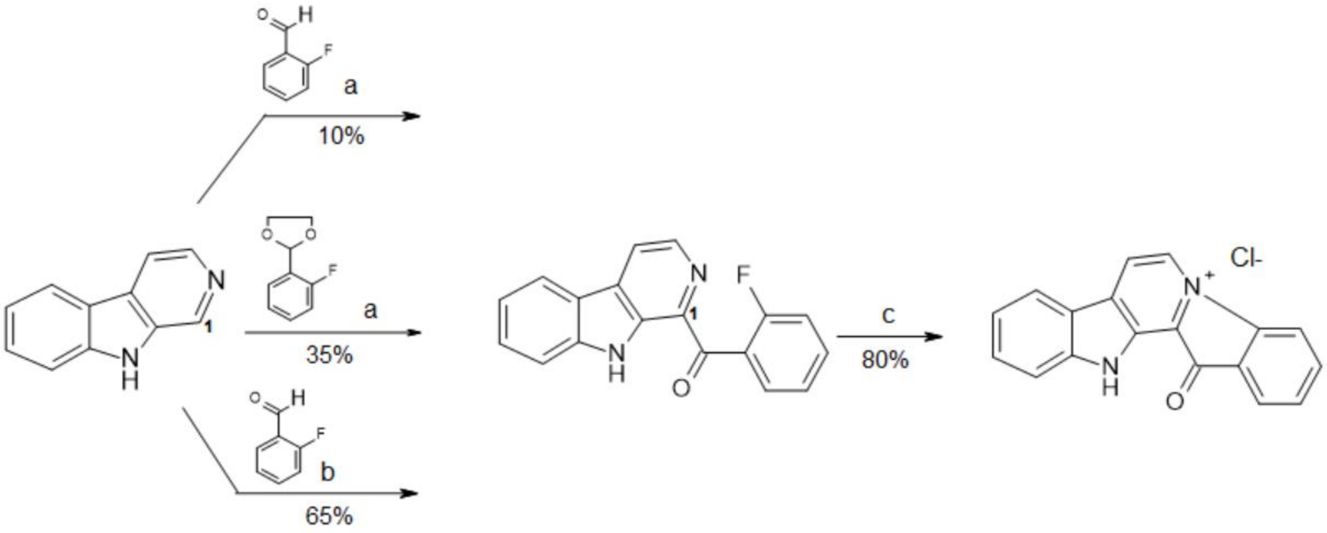

Reagents and conditions: (a) 70% *t*-BuOOH, $FeSO_4$ x $7H_2O$, CF_3COOH, CH_3COOH, H_2O, 4 °C, 30 min, twice; (b) 70% *t*-BuOOH, MW, 10 W, CF_3COOH, 40 min, several times; c) Py x HCl, 200-220 °C, 40 min.

Zhidkov, M.E.; Kaminskii, V.A. A new method for the synthesis of the marine alkaloid fascaplysin based on the mi-cro-wave-assisted Minisci reaction. Tetrahedron Lett. 2013, 54, 3530. https://doi.org/10.1016/j.tetlet.2013.04.113.

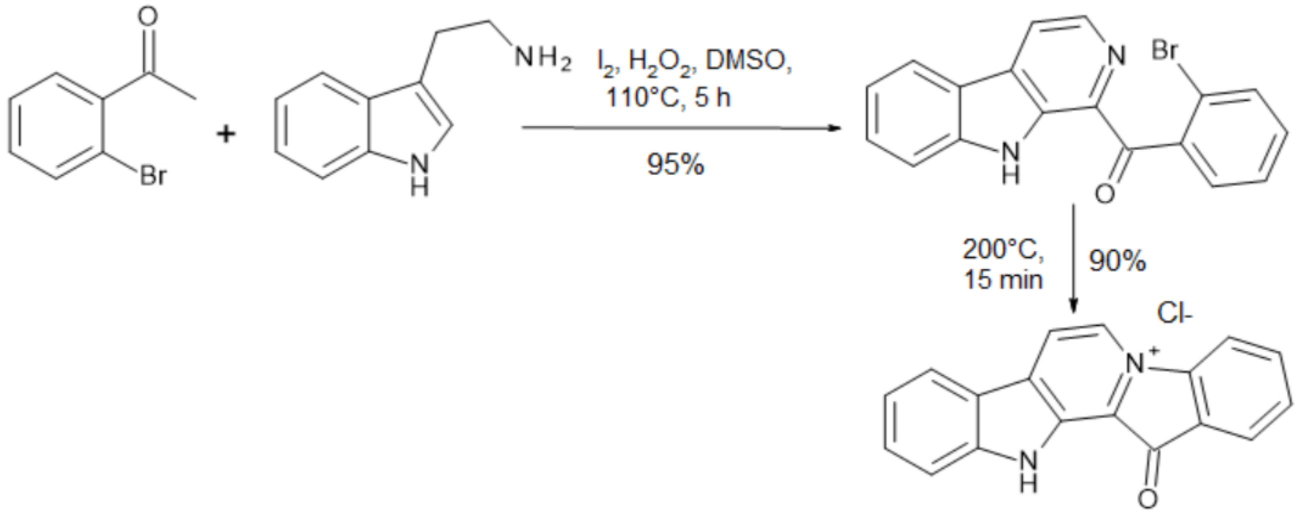

Zhu, Y.-P.; Liu, M.-C.; Cai, Q.; Jia, F.-C.; Wu, A.-X. A cascade coupling strategy for one-pot total synthesis of β-carboline and isoquinoline-containing natural products and derivatives. Chem. A Eur. J. 2013, 19, 10132. https://doi.org/10.1002/chem.201301734.

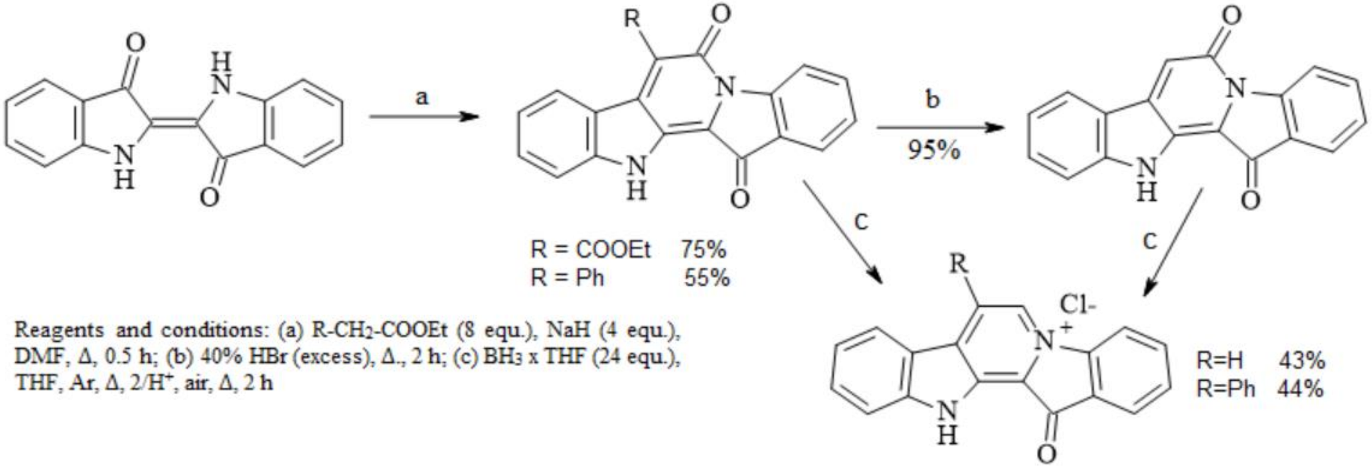

Zhidkov, M.E.; Kantemirov, A.V.; Koisevnikov, A.V.; Andin, A.N.; Kuzmich, A.S. Syntheses of the marine alkaloids 6-oxofascaplysin, fascaplysin and their derivatives. Tetrahedron Lett. 2018, 59, 708. https://doi.org/10.1016/j.tetlet.2018.01.023.

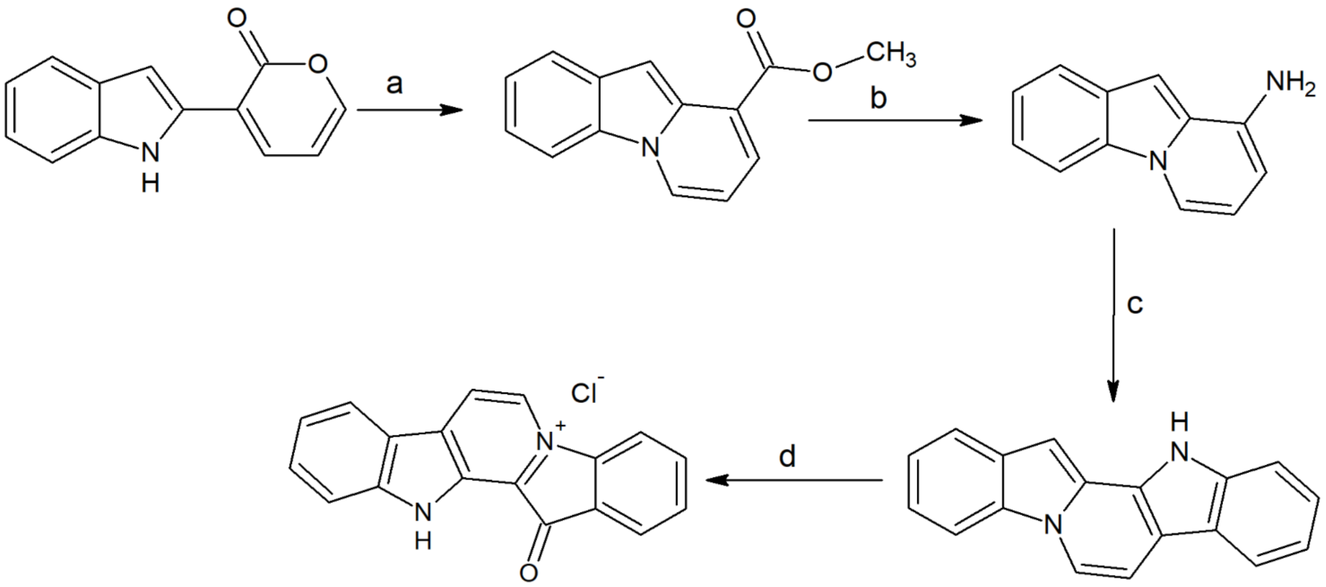

Reagents and conditions: (a) NaOMe, CH_2Cl_2/MeOH, r.t., 25 min, 65%; (b) KOH, 99%, DPPA, H_2O, 94%, (c) 1.2-dibromobenzole, $Pd(OAc)_2$, dppf, t-BuONa, 55%; (d) CH_3CO_3H, MeOH, 0 °C, 45 min, HCl, 85%.

Palani, V.; Perea, M.A.; Gardner, K.E.; Sarpong, R. A pyrone remodeling strategy to access diverse heterocycles: Application to the synthesis of fascaplysin natural products. Chem. Sci. 2021, 12, 1528. https://doi.org/10.1039/d0sc06317g

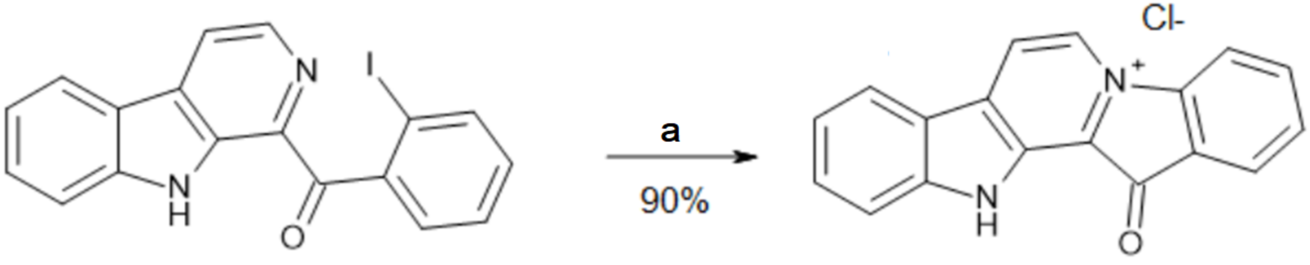

Reagents and conditions: (a) 1-(2'-iodobenzoyl)-β-carboline (19) 2 mM in acetonitrile, UV irradiation, -5 °C, 3x1.5 h

Tryapkin, O.A.; Kantemirov, A.V.; Dyshlovoy, S.A.; Prassolov, V.S.; Spirin, P.V.; von Amsberg, G.; Sidorova, M.A.; Zhidkov, M.E. A new mild method for synthesis of marine alkaloid fascaplysin and its therapeutically promising derivatives. Mar. Drugs 2023, 21, 424. https://doi.org/10.3390/md2108042 4
